# Phytochemical investigation on the aerial parts of *Veratrum versicolor* f. *viride* Nakai and their biological activities

**DOI:** 10.55730/1300-0527.3618

**Published:** 2023-09-12

**Authors:** Seong Su HONG, Jae Yeon LEE, Yeon Woo JEONG, Ji Eun LEE, Yun-Hyeok CHOI, Wonsik JEONG, Eun-Kyung AHN, Chun Whan CHOI, Il Ho AHN, Joa Sub OH

**Affiliations:** 1 Bio-Center, Gyeonggido Business & Science Accelerator (GBSA), Suwon, Republic of Korea; 2 NewCellPharm Co., Ltd., Seongnam, Republic of Korea; 3 College of Pharmacy, Dankook University, Cheonan, Republic of Korea

**Keywords:** *Veratrum*, flavonoid, stilbenoid, tyrosinase, elastase, melanogenesis

## Abstract

*Veratrum* spp. have traditionally been used in folk medicine to treat various pathologies. In this study, nine compounds, comprising one simple phenolic compound (**1**), three stilbenoids (**2**–**4**), and five flavonoids (**5**–**9**), were isolated from the aerial parts of *Veratrum versicolor* f. *viride* Nakai. The structures of these compounds were elucidated by spectroscopic analyses and comparison with reported data. Together, all reported compounds were isolated from *V. versicolor* f. *viride* for the first time in the study. Among them, two flavone aglycone tricetins (**7** and **9**) have never been isolated from the genus *Veratrum* or the family Melanthiaceae. The ethanol extract and isolated compounds were assessed for their inhibitory effects on elastase, tyrosinase, and melanin synthesis. Compounds **5** and **7** inhibited elastase (IC_50_: 292.25 ± 14.39 and 800.41 ± 5.86 μM, respectively), whereas compounds **2**–**5** inhibited tyrosinase with IC_50_ values in the range of 6.42 ~ 51.19 μM, respectively. In addition, compounds **3**–**6** and **8** exhibited dose-dependent inhibition (70.4% ~ 91.0%) of melanogenesis at a concentration of 100 μM.

## 1. Introduction

The genus *Veratrum* is a member of the family Melanthiaceae and consists of approximately 40 plant species distributed in temperate regions of the Northern Hemisphere, including Asia, Europe, and North America [1]. There are seven species (*V. coreanum* O.Loes., *V. dahuricum* O.Loes., *V. dolichopetalum* O.Loes., *V. maackii* Regel., *V. nigrum* L., *V. oxysepalum* Turcz., and *V. versicolor* Nakai), three varieties (*V. bohnhofii* var. *latifolium* Nakai, *V. maackii* var. *japonicum* (Baker) T.Shimizu, and *V. maackii* var. *parviflorum* (Maxim. ex Miq.) H.Hara), and two forms (*V. versicolor* f. *brunnea* Nakai and *V. versicolor* f. *viride* Nakai) in Korea that are widely spread across the Korean peninsula [2]. The rhizomes and roots of *V. nigrum* and *V. oxysepalum* have been used in traditional Korean medicine for centuries and are denoted as “Veratri Rhizoma et Radix” in the Korean Herbal Pharmacopoeia. These plants are indicated for the treatment of inflamed tonsils, snakebites, sore throats, coughs, dyspnea in epilepsy or stroke patients, and wrist pain [3]. Several species of *Veratrum* (*V. album* L., *V. californicum* Durand, *V. viride* Röhl., and *V. nigrum* var. *japonicum* (Baker) T.Shimizu) are harmful to humans and animals, and earlier research has shown that steroidal alkaloid constituents are responsible for this toxicity [4]. *Veratrum* species are rich sources of plant-derived steroidal alkaloids [5,6]. Among the various types of steroidal alkaloids, veratrum-type steroidal alkaloids, which are the most frequently found in the genus *Veratrum*, can be categorized into five subtypes: cevanine, veratramine, jervine, solanidine, and verazine [6]. Moreover, many of these *Veratrum* alkaloids have been reported to possess a range of biological activities, such as antiproliferative [5], antidiabetic [7], anticancer [8], antifungal [9], antihypertensive [10], antiinflammatory [11], antioxidant [12], and potent analgesic [13,14] properties. Previous phytochemical investigations of the chemical constituent species in *Veratrum* have identified a diverse range of compounds, including arylbenzofurans [15], cevanine-type alkaloids [16], veratramine-type alkaloids [14], jervine-type alkaloids [12,17], solanidine-type alkaloids [18], verazine-type alkaloids [19], flavonoids [20], stilbene glycosides [21], and aurones [22]. To date, most studies on *Veratrum* spp. have focused on steroidal alkaloids. However, to the best of our knowledge, no previous investigations have been conducted on the phytochemical and pharmacological properties of the constituents of *V. versicolor* f. *viride*.

## 2. Materials and methods

### 2.1. General

Nuclear magnetic resonance (NMR) spectra were recorded on a Bruker Ascend III 700 spectrometer (Bruker BioSpin GmbH., Rheinstetten, Germany) in DMSO-*d_6_* and CD_3_OD at room temperature. Chemical shifts are in ppm (δ), relative to tetramethylsilane as an internal standard, and coupling constants are in hertz; ^1^H, ^13^C, DEPT, COSY, HSQC, and HMBC were performed using the standard pulse sequences. Electrospray ionization mass (ESI-MS) spectra were acquired on an Agilent 6130 series quadrupole LC/MS System (Agilent Technologies, Santa Clara, CA, USA). Open column chromatography was performed using Diaion HP-20 adsorbent resin (Mitsubishi Chemical Corp, Tokyo, Japan). Medium-pressure liquid chromatography (MPLC) was conducted using a CombiFlash Rf flash chromatography system (Teledyne ISCO Inc., Lincoln, NE, USA), and the separations were performed on a Redi*Sep*^®^ Rf C_18_ column with a flow rate of 40 mL/min. Preparative high-performance liquid chromatography (HPLC) was performed on a Thermo Scientifc Dionex Ultimate 3000 UHPLC system (Thermo Fisher Scientific Inc., Waltham, MA, USA) equipped with an HPG-3200BX biocompatible binary semipreparative pump and a rapid separations PDA detector (Ultimate DAD-3000) controlled by Chromeleon 7.2 software. The separations were carried out on a Kromasil 100-5-C18 column (5 μm, 21.2 × 250 mm, Nouryon Chemicals Finance B.V., Amsterdam, Netherlands). Thin layer chromatography (TLC) was performed using DC-Fertigfolien ALUGRAM SIL G/UV_254+366_ (0.2 mm, Macherey-Nagel GmbH & Co. KG, Düren, Germany) plates, and spots were visualized by a 10% vanillin–sulfuric acid reagent. All chemicals and solvents were of analytical grade and were used without further purification.

### 2.2. Plant material

The aerial parts of *V. versicolor* f. *viride* Nakai [23] were collected from Cheorwon-gun, Gangwon-do, Republic of Korea, in July 2022. The collection area is 868 m above sea level and located at GPS 38°8′30.87″N 127°26′22.95″E and they grow naturally at the edge of the ridge. Organic matter in soil is moderate (2%–4%) and it is black forest soil. The crown density is 80% and the quantity of light is poor. The botanical materials were authenticated by one of the authors (Prof. J.S. Oh) and a voucher specimen (G105) was deposited at the Bio-Center, Gyeonggido Business & Science Accelerator (GBSA), Suwon, Republic of Korea.

### 2.3. Extraction and isolation

**Figure 1:**
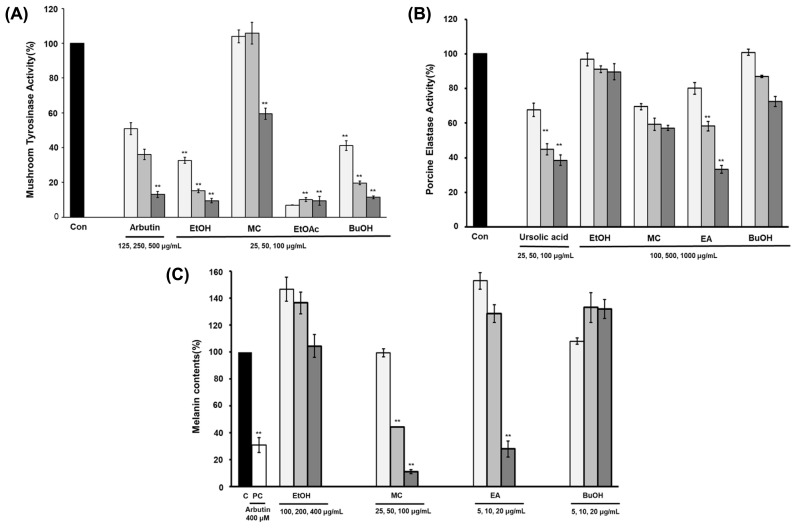
Tyrosinase (A), elastase (B), and melanogenesis (C) inhibitory activity of extracts obtained from aerial parts of *V. versicolor* f. *viride*. The asterisks represent a significant difference between the columns. **, p < 0.01. The bars represent standard errors.

The shade-dried aerial parts of *V. versicolor* f. *viride* (2 kg) were percolated with 70% aqueous EtOH at room temperature. Following evaporation of the solvent under reduced pressure, the residue (265 g) was suspended in water and successively partitioned with CH_2_Cl_2_, EtOAc, and water-saturated *n*-BuOH to yield the respective extracts (17 g, 12.5 g, and 24 g) after being concentrated to dryness. The crude extracts and solvent layers of *V. versicolor* f. *viride* were screened for their inhibitory effects on tyrosinase, elastase, and melanogenesis at various concentrations (5 ~ 1000 μg/mL) (Figure 1). The results showed that the EtOAc layer exhibited dose-dependent inhibitory activity. Part of the EtOAc-soluble fraction was chromatographed over a Diaion HP-20 resin and eluted with a water–MeOH stepwise gradient solvent system (1:0 to 0:1) to yield five fractions (G105B_1_–G105B_5_). Fraction G105B_3_ (1.5 g) was subjected to MPLC over an ODS column (RediSep Rf silica gold 150 g, 40 mL/min, 120 min) by eluting with a gradient mixture of 10% ~ 35% acetonitrile in water to yield compounds 1 (16.2 mg, *t*_R_ = 25.2 min), **2** (45.3 mg, *t*_R_ = 41.1 min), 3 (96.4 mg, *t*_R_ = 47.4 min), and 4 (73.1 mg, *t*_R_ = 71.4 min). Subfraction G105B_4_ (86.9 mg) was subjected to the above MPLC system (acetonitrile–H_2_O, 20:80 to 40:60, v/v), resulting in the isolation of compounds **5** (36.6 mg, *t*_R_ = 37.8 min) and 9 (1.3 mg, *t*_R_ = 42.8 min). Compound 6 (15.1 mg, *t*_R_ = 78.1 min) and two compound mixtures (G105C_4_, 15.1 mg, *t*_R_ = 81.5 min) were isolated from subfraction G105B_5_ using MPLC (acetonitrile–water, 15:85 to 45:55 in 120 min). Further purification of subfraction G105C_4_ was performed using preparative HPLC (Kromasil 100-5-C18 column; 21.2 × 250 mm; flow rate, 10 mL/min; solvent A, 0.05% TFA in water; solvent B, acetonitrile; gradient elution 0 min 30% B to 120 min 40% B, detection at 210 and 350 nm). HPLC separation led to purification of compounds 7 (36.2 mg, t_R_ = 38.2 min) and 8 (11.2 mg, *t*_R_ = 41.8 min). The isolation process used in the present study is summarized in Figure S1 (Supporting Information).

### 2.4. Spectral data of isolated compounds

The structures of the isolated compounds were elucidated by MS and 1D/2D NMR data analyses and compared with the corresponding data reported in the literature.

**Vanillic acid (1):** White amorphous powder, ^1^H NMR (CD_3_OD, 700 MHz): δ 7.56 (1H, dd, *J* = 8.4, 2.1 Hz, H-6), 7.56 (1H, d, *J* = 2.1 Hz, H-2), 6.84 (1H, d, *J* = 8.4 Hz, H-5), 3.89 (3H, s, 3-OCH_3_); ^13^C NMR (CD_3_OD, 175 MHz): δ 170.2 (C-7), 152.8 (C-4), 148.8 (C-3), 123.3 (C-1), 125.4 (C-6), 116.0 (C-5), 113.9 (C-2), 56.4 (3-OCH_3_); UV (CH_3_OH) λ_max_ nm: 204, 218, 260, 291; ESIMS (positive ion mode) *m/z* 169 [M + H]^+^; (Supporting Information, Figures S2 and S3) [24].

***trans*-Piceid (2):** Pale brown amorphous powder, ^1^H NMR (CD_3_OD, 700 MHz): δ 7.36 (2H, d, *J* = 9.1 Hz, H-2′, 6′), 7.02 (1H, d, *J* = 16.1 Hz, H-8), 6.85 (1H, d, *J* = 16.1 Hz, H-7), 6.76 (2H, d, *J* = 9.1 Hz, H-3′, 5′), 6.79 (1H, t, *J* = 2.1 Hz, H-2), 6.61 (1H, t, *J* = 2.1 Hz, H-6), 6.45 (1H, t, *J* = 2.1 Hz, H-4), 4.89 (1H, d, *J* = 7.7 Hz, H-1″), 3.93 (1H, dd, *J* = 11.9, 2.1 Hz, H_a_-6″), 3.71 (1H, dd, *J* = 11.9, 5.6 Hz, H_b_-6″), 3.47 (1H, t, *J* = 9.1 Hz, H-5″), 3.46 (1H, m, H-3″), 3.45 (1H, t, *J* = 9.1 Hz, H-2″), 3.38 (1H, t, *J* = 9.1 Hz, H-4″); ^13^C NMR (CD_3_OD, 175 MHz): δ 160.6 (C-3), 159.7 (C-5), 158.6 (C-4′), 141.6 (C-1), 130.5 (C-1′), 130.1 (C-8), 129.1 (C-2′, 6′), 126.8 (C-7), 116.2 (C-3′, 5′), 108.5 (C-6), 107.1 (C-2), 104.2 (C-4), 102.5 (C-1″), 78.4 (C-5″), 78.2 (C-3″), 75.1 (C-2″), 71.6 (C-4″), 62.7 (C-6″); UV (CH_3_OH) λ_max_ nm: 214, 233 (sh), 306, 319; ESIMS (positive ion mode) *m/z* 391 [M + H]^+^; (Supporting Information, Figures S4 and S5) [25].

**Oxyresveratrol (3):** Pale brown amorphous powder, ^1^H NMR (CD_3_OD, 700 MHz): δ 7.32 (1H, d, *J* = 8.4 Hz, H-6′), 7.26 (1H, d, *J* = 16.1 Hz, H-8), 6.81 (1H, d, *J* = 16.1 Hz, H-7), 6.44 (2H, d, *J* = 2.1 Hz, H-2, 6), 6.30 (1H, dd, *J* = 8.4, 2.1 Hz, H-5′), 6.29 (1H, d, *J* = 2.1 Hz, H-3′), 6.13 (1H, t, *J* = 2.1 Hz, H-4); ^13^C NMR (CD_3_OD, 175 MHz): δ 159.6 (C-3, 5), 159.4 (C-4′), 157.5 (C-2′), 142.2 (C-1), 118.0 (C-1′), 124.9 (C-8), 128.5 (C-6′), 126.6 (C-7), 108.5 (C-5′), 103.7 (C-3′), 105.8 (C-2, 6), 102.4 (C-4); UV (CH_3_OH) λ_max_ nm: 217, 239 (sh), 301, 327; ESIMS (positive ion mode) *m/z* 245 [M + H]^+^; (Supporting Information, Figures S6 and S7) [26].

**Resveratrol (4):** Pale brown amorphous powder, ^1^H NMR (CD_3_OD, 700 MHz): δ 7.35 (2H, d, *J* = 8.4 Hz, H-2′, 6′), 6.95 (1H, d, *J* = 16.1 Hz, H-8), 6.80 (1H, d, *J* = 16.1 Hz, H-7), 6.76 (2H, d, *J* = 8.4 Hz, H-3′, 5′), 6.45 (2H, d, *J* = 2.1 Hz, H-2, 6), 6.16 (1H, t, *J* = 2.1 Hz, H-4); ^13^C NMR (CD_3_OD, 175 MHz): δ 159.7 (C-3, 5), 158.5 (C-4′), 141.3 (C-1), 130.6 (C-1′), 129.5 (C-8), 128.9 (C-2′, 6′), 127.1 (C-7), 116.6 (C-3′, 5′), 105.9 (C-2, 6), 102.8 (C-4); UV (CH_3_OH) λ_max_ nm: 216, 237 (sh), 304, 319; ESIMS (positive ion mode) *m/z* 229 [M + H]^+^; (Supporting Information, Figures S8 and S9) [27].

**Luteolin (5):** Yellow amorphous powder, ^1^H NMR (DMSO-*d_6_*, 700 MHz): δ 12.99 (1H, s, 5-OH), 10.83 (1H, s, 7-OH), 9.93 (1H, s, 4′-OH), 9.41 (1H, s, 3′-OH), 7.42 (1H, dd, *J* = 8.4, 2.1 Hz, H-6′), 7.40 (1H, d, *J* = 2.1 Hz, H-2′), 6.90 (1H, d, *J* = 8.4 Hz, H-5′), 6.78 (1H, s, H-3), 6.45 (1H, d, *J* = 2.1 Hz, H-8), 6.20 (1H, d, *J* = 2.1 Hz, H-6); ^13^C NMR (DMSO-*d_6_*, 175 MHz): δ 181.7 (C-4), 164.1 (C-7), 163.9 (C-2), 161.5 (C-5), 157.3 (C-9), 149.7 (C-4′), 145.7 (C-3′), 121.5 (C-1′), 119.0 (C-6′), 116.0 (C-5′), 113.4 (C-2′), 103.7 (C-10), 102.9 (C-3), 98.8 (C-6), 93.8 (C-8); UV (CH_3_OH) λ_max_ nm: 206, 252, 269, 347; ESIMS (positive ion mode) *m/z* 289 [M + H]^+^; (Supporting Information, Figures S10 and S11) [28].

**Apigenin (6):** Yellow amorphous powder, ^1^H NMR (DMSO-*d_6_*, 700 MHz): δ 12.97 (1H, s, 5-OH), 10.85 (1H, s, 7-OH), 10.37 (1H, s, 4′-OH), 7.93 (2H, d, *J* = 8.4 Hz, H-2′, 6′), 6.93 (2H, d, *J* = 8.4 Hz, H-3′, 5′), 6.79 (1H, s, H-3), 6.49 (1H, d, *J* = 2.1 Hz, H-8), 6.20 (1H, d, *J* = 2.1 Hz, H-6); ^13^C NMR (DMSO-*d_6_*, 175 MHz): δ 181.8 (C-4), 164.1 (C-7), 163.7 (C-2), 161.5 (C-5), 161.2 (C-4′), 157.3 (C-9), 128.5 (C-2′, 6′), 121.2 (C-1′), 116.0 (C-3′, 5′), 103.7 (C-10), 102.8 (C-3), 98.8 (C-6), 94.0 (C-8); UV (CH_3_OH) λ_max_ nm: 207, 266, 337; ESIMS (positive ion mode) *m/z* 271 [M + H]^+^; (Supporting Information, Figures S12 and S13) [28].

**Tricin (7):** Yellow amorphous powder, ^1^H NMR (DMSO-*d_6_*, 700 MHz): δ 12.97 (1H, s, 5-OH), 10.81 (1H, s, 7-OH), 9.34 (1H, s, 4′-OH), 7.32 (2H, s, H-2′, 6′), 6.98 (1H, s, H-3), 6.56 (1H, d, *J* = 2.1 Hz, H-8), 6.20 (1H, d, *J* = 2.1 Hz, H-6), 3.88 (6H, s, 3′, 5′-OCH_3_); ^13^C NMR (DMSO-*d_6_*, 175 MHz): δ 181.8 (C-4), 164.1 (C-7), 163.7 (C-2), 161.4 (C-5), 157.3 (C-9), 148.2 (C-3′, 5′), 139.8 (C-4′), 120.4 (C-1′), 104.3 (C-2′, 6′), 103.7 (C-10), 103.6 (C-3), 98.8 (C-6), 94.2 (C-8), 56.4 (3′,5′-OCH_3_); UV (CH_3_OH) λ_max_ nm: 209, 243 (sh), 270, 351; ESIMS (positive ion mode) *m/z* 331 [M + H]^+^; (Supporting Information, Figures S14 and S15) [29].

**Chrysoeriol (8):** Yellow amorphous powder, ^1^H NMR (DMSO-*d_6_*, 700 MHz): δ 12.98 (1H, s, 5-OH), 10.86 (1H, s, 7-OH), 9.99 (1H, s, 4′-OH), 7.57 (1H, dd, *J* = 8.4, 2.1 Hz, H-6′), 7.56 (1H, d, *J* = 2.1 Hz, H-2′), 6.91 (1H, s, H-3), 6.84 (1H, d, *J* = 8.4 Hz, H-5′), 6.52 (1H, d, *J* = 2.1 Hz, H-8), 6.20 (1H, d, *J* = 2.1 Hz, H-6), 3.90 (3H, s, 3′-OCH_3_); ^13^C NMR (DMSO-*d_6_*, 175 MHz): δ 181.8 (C-4), 164.1 (C-7), 163.7 (C-2), 161.4 (C-5), 157.3 (C-9), 150.7 (C-4′), 148.0 (C-3′), 121.5 (C-1′), 120.4 (C-6′), 115.8 (C-5′), 110.2 (C-2′), 103.7 (C-10), 103.2 (C-3), 98.8 (C-6), 94.1 (C-8), 56.0 (3′-OCH_3_); UV (CH_3_OH) λ_max_ nm: 206, 250, 266, 346; ESIMS (positive ion mode) *m/z* 301 [M + H]^+^; (Supporting Information, Figures S16 and S17) [30].

**3**′***-O*-Methyltricetin (9):** Yellow amorphous powder, ^1^H NMR (DMSO-*d_6_*, 700 MHz): δ 13.09 (1H, s, 5-OH), 10.79 (1H, s, 7-OH), 9.42 (1H, s, 5′-OH), 9.25 (1H, s, 4′-OH), 7.17 (1H, d, *J* = 2.1 Hz, H-2′), 7.15 (1H, d, *J* = 2.1 Hz, H-6′), 6.83 (1H, s, H-3), 6.47 (1H, d, *J* = 2.1 Hz, H-8), 6.20 (1H, d, *J* = 2.1 Hz, H-6), 3.88 (3H, s, 3′-OCH_3_); ^13^C NMR (DMSO-*d_6_*, 175 MHz): δ 181.7 (C-4), 164.0 (C-7), 163.9 (C-2), 161.4 (C-5), 157.3 (C-9), 148.6 (C-3’), 145.9 (C-5′), 138.6 (C-4′), 120.4 (C-1′), 107.5 (C-6′), 103.7 (C-10), 103.3 (C-3), 102.4 (C-2′), 98.8 (C-6), 93.9 (C-8), 56.2 (3′-OCH_3_); UV (CH_3_OH) λ_max_ nm: 209, 266, 352; ESIMS (positive ion mode) *m/z* 317 [M + H]^+^; (Supporting Information, Figures S18 and S19) [31].

### 2.5. Biological assay

#### 2.5.1. Tyrosinase inhibition assay

The reaction was carried out in a 0.1 M potassium phosphate buffer (pH 6.5) containing 1.5 mM L-tyrosine and 1250 unit/mL mushroom tyrosinase and the reaction mixture was incubated at 37 °C for 20 min. The test samples were assayed for tyrosinase inhibition by measuring its effect on tyrosinase activity using an ELISA reader at 490 nm. Arbutin was used as a positive control. The inhibitory activity of the sample was expressed as the concentration that inhibits 50% of the enzyme activity (IC_50_) [32].

#### 2.5.2. Elastase inhibition assay

The reaction was carried out in a 50 mM Tris-HCl buffer (pH 8.5) containing 1 mg/mL N-succinyl-(Ala)3-p-nitroanilide and 0.6 U/mL PPE (porcine pancreas elastase). The test sample was added to the reaction mixture, and elastase inhibition was incubated at 25 °C for 10 min. The change in absorbance was measured at 405 nm using an ELISA reader. Ursolic acid was used as a positive control. The inhibitory activity of the sample was expressed as the concentration that inhibits 50% of the enzyme activity (IC_50_) [33,34].

#### 2.5.3. Cell culture

B16F10 mouse melanoma cells were cultured in DMEM medium containing 10% FBS and 1% penicillin-streptomycin in a 37 °C incubator with 5% CO_2_. The cells were seeded in 100-mm tissue culture dishes to be used for subsequent experiments [32].

#### 2.5.4. Measurement of relative melanin contents

B16F10 cells (1 × 10^5^/well) were seeded in 6 well plates for 24 h. The cells were then incubated in the presence of 100 nM α-MSH, and treated with various concentrations (25, 50, and 100 μM) of sample for 72 h. After being washed twice with PBS, the cells were dissolved of 1 N NaOH and 10% DMSO, incubated at 60 °C for 1 h, and mixed to solubilize the melanin. Relative melanin content was determined with an ELISA reader by absorbance at 405 nm [32].

#### 2.5.5. Statistical analysis

All data are presented as means ± standard deviation (SD). The results were analyzed for statistical significance using Student’s t-test and one-way analysis of variance (ANOVA). Values of *p < 0.05 and **p < 0.01 were considered statistically significant.

## 3. Results and discussion

**Figure 2:**
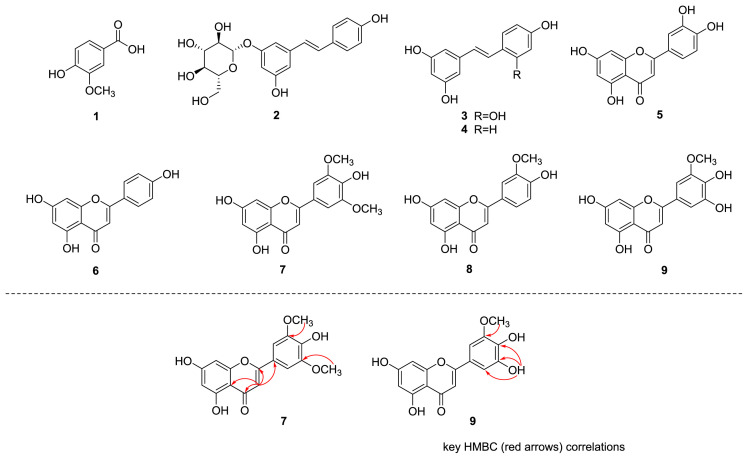
Chemical structures of compounds **1**–**9** identified from *V. versicolor* f. *viride*.

In the present study, nine compounds were isolated from the aerial parts of *V. versicolor* f. *viride*, comprising one phenolic analog (1), three stilbenoids (2–4), and five flavonoids (5–9) (Figure 2). The compounds isolated were identified as vanillic acid (1), *trans*-piceid (2), oxyresveratrol (3), resveratrol (4), luteolin (5), apigenin (6), and chrysoeriol (8) by comparing their spectroscopic data with reference values from previously published literature (Figure 2).

Compound 7 was obtained as a pale yellow amorphous powder. The UV spectrum showed maximum absorption bands of a flavonoid system at λ_max_ 351, 270, and 209 nm. The ^1^H NMR data showed the presence of two *meta*-coupled aromatic protons [δ_H_ 6.56 1H, d, *J* = 2.1 Hz, H-8), 6.20 (1H, d, *J* = 2.1 Hz, H-6)], a symmetrical 1,3,4,5-tetrasubstitution of the benzene ring [δ_H_ 7.32 (2H, s, H-2′, H-6′)], one isolated aromatic proton [δ_H_ 6.98 (1H, s, H-3)], and two methoxyls [δ_H_ 3.88 (6H, s)]. Combined with the ^13^C NMR, the DEPT and HSQC spectra of **7** exhibited resonances for 17 signals, including one conjugated carbonyl carbon (δ_C_ 181.8), seven oxygenated sp^2^ carbons (δ_C_ 164.1, 163.7, 161.4, 157.3, 148.2 × 2, and 139.8), two quaternary carbons (δ_C_ 120.4 and 103.7), five aromatic methine carbons (δ_C_ 104.3 × 2, 103.6, 98.8, and 94.2), and two methoxy groups (δ_C_ 56.4 × 2). A comparison of the NMR data of 7 with those of tricetin (flavonoid) [35] revealed the presence of an additional methoxy group, suggesting that 7 was the methylated derivative of tricetin. The HMBC experiment of 7 showed correlations between methoxy signal (δ_H_ 3.88)/C-3′ and C-5′ (δ_C_ 148.2), confirming the substitution of the methoxyl group at C-3′ and C-5′ (Figure 2). Consequently, the structure of **7** was established to be 4′,5,7-trihydroxy-3′,5′-dimethoxyflavone (tricin) [29].

**Figure 3:**
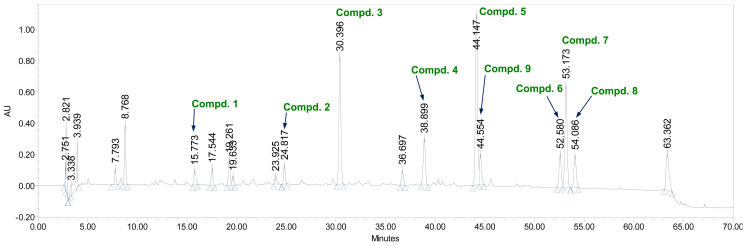
HPLC–PDA chromatographic profile of the EtOAc-soluble extract from *Veratrum versicolor* f. *viride* aerial parts was recorded at 210 nm. Column, YMC-Triart C18 (250 × 4.6 mm I.D., 5 μm); mobile phase, 0.05% trifluoroacetic acid in water (A) and acetonitrile (B), initial 90:10 (A:B, v/v), 60 min 60:40 (A:B, v/v); flow rate, 1 mL/min.

The structure of 9 was closely related to 7 based on UV and ^1^H and ^13^C NMR spectroscopic data. However, 9 had one carbon and two protons less than **7**. The appearance of a sharp singlet at δ_H_ 9.42 in the ^1^H NMR spectrum of 9 and the absence of the signals of a methoxy group and a methoxy carbon in the ^1^H and ^13^C NMR spectra of 9, respectively, suggested a hydrogen bonded phenolic hydroxyl group at C-5′. The HMBC experiment of 9 showed correlations between 5′-OH (δ_H_ 9.42)/C-5′ (δ_C_ 145.9), C-4′ (δ_C_ 138.6) and C-6′ (δ_C_ 107.5), confirming the substitution of the phenolic hydroxyl group at C-5′ (Figure 2). On the basis of this evidence, the structure of the molecule was confirmed as 4′,5′,5,7-tetrahydroxy-3′ methoxyflavone (3′-*O*-methyltricetin) [31]. To the best of our knowledge, this is the first report of all aforementioned compounds being isolated from this plant, as well as compounds 7 and 9 from the family Melanthiaceae. The HPLC profiles of the isolated compounds are shown in Figure 3.

**Figure 4:**
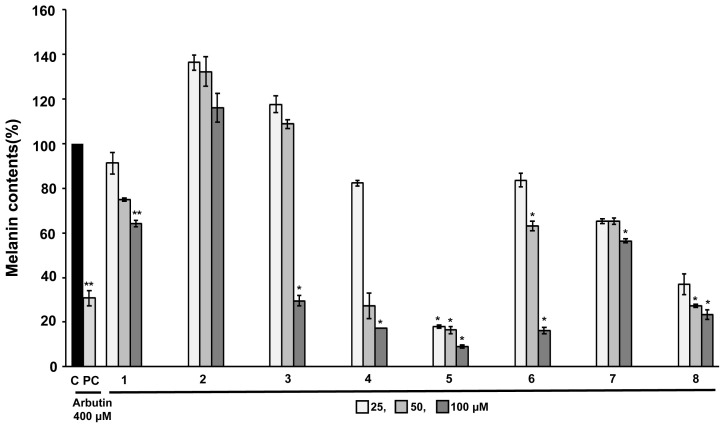
Effects of isolated compounds and arbutin (positive control) on melanin production in B16F10 mouse melanoma cells. The data are expressed as the mean value ± standard deviation of three independent experiments (*, p < 0.05).

The tyrosinase inhibitory effects of the isolated compounds were examined, and IC_50_ was calculated using a dose-dependent response curve. The IC_50_ values for compounds 2–5 were as follows: 51.19 ± 5.50, 6.42 ± 0.45, 19.82 ± 0.77, and 37.89 ± 1.04 μM, respectively (Table). The IC_50_ values of arbutin (tyrosinase inhibitor) as a positive control were 473.65 μM [36]. With this bioassay, no tyrosinase inhibitory activity (IC_50_ > 100 M) was detected for other compounds. Moreover, inhibition of elastase enzyme (porcine pancreas) activity was used to evaluate the antiaging properties of the isolated compounds, and the results are shown in the Table. It was observed that compounds 5 and 8 showed moderate antielastase activity with IC_50_ values of 292.25 ± 14.39 and 800.41 ± 5.86 μM, respectively, compared to ursolic acid [37] (IC_50_: 96.88 μM), which was used as the positive control. A melanin content assay was conducted to observe the inhibitory effect of the isolated constituents on melanin production. Here B16F10 melanoma cells were stimulated by α-MSH and cotreated with compounds in three different concentrations (25, 50, and 100 μM) or arbutin (400 μM). According to the results shown in Figure 4, compounds 4, 5, and 8 greatly decreased the melanin content in α-MSH-stimulated B16F10 melanoma cells at 100 μM, as compared to those of arbutin. In the range of 50 ~ 100 μM, the inhibition percentages of resveratrol (4), luteolin (**5**), and chrysoeriol (8) were 72.7% ~ 82.7%, 83.5% ~ 91.0%, and 72.6% ~ 76.6%, respectively, whereas the inhibition rate of 400 μM arbutin was 69.2% (Supporting Information, Table S1) [38]. In conclusion, extracts and active compounds effectively inhibited elastase and mushroom tyrosinase activities and inhibited melanin synthesis by B16F10 melanoma cells. Thus, *V. versicolor* f. *viride* extracts and several of their chemical constituents can be considered useful for application in developing multitarget cosmeceuticals.

## 4. Conclusion

As part of our ongoing work in the search for biologically active constituents from Korean resource plants, an EtOH extract of *V. versicolor* f. *viride* was selected for phytochemical investigation. Nine natural compounds were isolated from the aerial parts of *V. versicolor* f. *viride*, comprising one phenolic analog (1), three stilbenoids (2–4), and five flavonoids (5–9), and the effectiveness of their biological potential was demonstrated. Based on enzyme and cell inhibitory assays, the isolated compounds could exhibit good antityrosinase, antielastase properties, and melanogenesis in B16F10 melanoma cells, thereby conferring a comprehensive attenuating effect against skin aging-related factors. Hence, the results of our study suggest that *V. versicolor* f. *viride* has great potential to be used as an effective multifunctional bioactive agent for cosmeceutical formulations. However, additional in-depth studies and clinical evaluations are needed to estimate the skincare potential of extracts and active compounds.

## Supporting Information

Figure S1 The isolation scheme of compounds **1**–**9**.

### 1. Apparatus and chromatographic conditions

HPLC analysis was performed on a Waters alliance e2695 (Waters Co., Milford, MA, USA) system composed of a 2998 PDA detector and column heater/cooler with a passive preheater. The separation was achieved using a YMC-Triart C18 column (250 × 4.6 mm I.D., 5 μm particle size) (YMC Co., Ltd., Japan). The mobile phase consisted of water–TFA (99.95:0.05; v/v) (solvent A) and acetonitrile (solvent B). The elution was performed using the following gradient: initial 90:10 (A:B v/v); 60 min 60:40 (A:B v/v). The mobile phase was prepared daily, filtered through a 0.45-mm, WTP 0.5-mm membrane (Whatmann, Maidstone, UK), sonicated before use and delivered at a flow rate of 1.0 mL/min. The injection volume was 10 μL and the column temperature was at 25 °C. All the operations, the acquiring and analysis of data were controlled by Empower 3 Software (Waters Co., Milford, MA, USA).

**Table S1:** Inhibitory activity (melanin contents) of compounds isolated from *V. versicolor* f. *viride* on melanogenesis in B16 mouse melanoma cells.

Compound	Melanin contents (%)			
	25 μM	50 μM	100 μM	400 μM
**1**	91.30 ± 4.81 ^a^	74.78 ± 0.69	64.01 ± 1.45	
**2**	136.33 ± 3.36	132.31 ± 6.52	116.14 ± 6.39	
**3**	117.63 ± 3.73	108.76 ± 1.96	29.56 ± 2.21	
**4**	82.28 ± 1.18	27.29 ± 5.83	17.30 ± 0.01	
**5**	17.98 ± 0.76	16.41 ± 1.65	8.99 ± 0.73	
**6**	83.60 ± 3.01	63.16 ± 2.11	16.24 ± 1.52	
**7**	65.25 ± 1.18	65.37 ± 1.41	56.39 ± 0.89	
**8**	37.02 ± 4.57	27.32 ± 0.80	23.36 ± 2.19	
Arbutin ^b^				30.71 ± 3.34

a All compounds were examined in a set of experiments three times.

b Positive control.

Figure S2 ^1^H NMR spectrum (CD_3_OD, 700 MHz) of compound **1**.

Figure S3 ^13^C NMR spectrum (CD_3_OD, 175 MHz) of compound **1**.

Figure S4 ^1^H NMR spectrum (CD_3_OD, 700 MHz) of compound **2**.

Figure S5 ^13^C NMR spectrum (CD_3_OD, 175 MHz) of compound **2**.

Figure S6 ^1^H NMR spectrum (CD_3_OD, 700 MHz) of compound **3**.

Figure S7 ^13^C NMR spectrum (CD_3_OD, 175 MHz) of compound **3**.

Figure S8 ^1^H NMR spectrum (CD_3_OD, 700 MHz) of compound **4**.

Figure S9 ^13^C NMR spectrum (CD_3_OD, 175 MHz) of compound **4**.

Figure S10 ^1^H NMR spectrum (DMSO-*d_6_*, 700 MHz) of compound **5**.

Figure S11 ^13^C NMR spectrum (DMSO-*d_6_*, 175 MHz) of compound **5**.

Figure S12 ^1^H NMR spectrum (DMSO-*d_6_*, 700 MHz) of compound **6**.

Figure S13 ^13^C NMR spectrum (DMSO-*d_6_*, 175 MHz) of compound **6**.

Figure S14 ^1^H NMR spectrum (DMSO-*d_6_*, 700 MHz) of compound **7**.

Figure S15 ^13^C NMR spectrum (DMSO-*d_6_*, 175 MHz) of compound **7**.

Figure S16 ^1^H NMR spectrum (DMSO-*d_6_*, 700 MHz) of compound **8**.

Figure S17 ^13^C NMR spectrum (DMSO-*d_6_*, 175 MHz) of compound **8**.

Figure S18 ^1^H NMR spectrum (DMSO-*d_6_*, 700 MHz) of compound **9**.

Figure S19 ^13^C NMR spectrum (DMSO-*d_6_*, 175 MHz) of compound **9**.

## Figures and Tables

**Table:** In vitro tyrosinase and elastase inhibitory activity of the isolated compounds.

Compound	Tyrosinase inhibitory activity	Elastase inhibitory activity
	IC_50_^a^ (μM)	
**1**	>100	>1000
**2**	51.19 ± 5.50	>1000
**3**	6.42 ± 0.45	>1000
**4**	19.82 ± 0.77	>1000
**5**	37.89 ± 1.04	292.25 ± 14.39
**6**	>100	>1000
**7**	>100	>1000
**8**	>100	800.41 ± 5.86
Arbutin^b^	473.65 ± 18.21	–
Ursolic acid^b^	–	96.88 ± 1.33

a All compounds were examined in a set of experiments three times.

b Positive control.
