# Supplementary figures and images for: Phytochemical investigation on the aerial parts of Veratrum versicolor f. viride Nakai and their biological activities
Source: Turk J Chem. 2023 Sep 12;47(6):1346–54. doi: 10.55730/1300-0527.3618 (PMC10965189; doi:10.55730/1300-0527.3618)

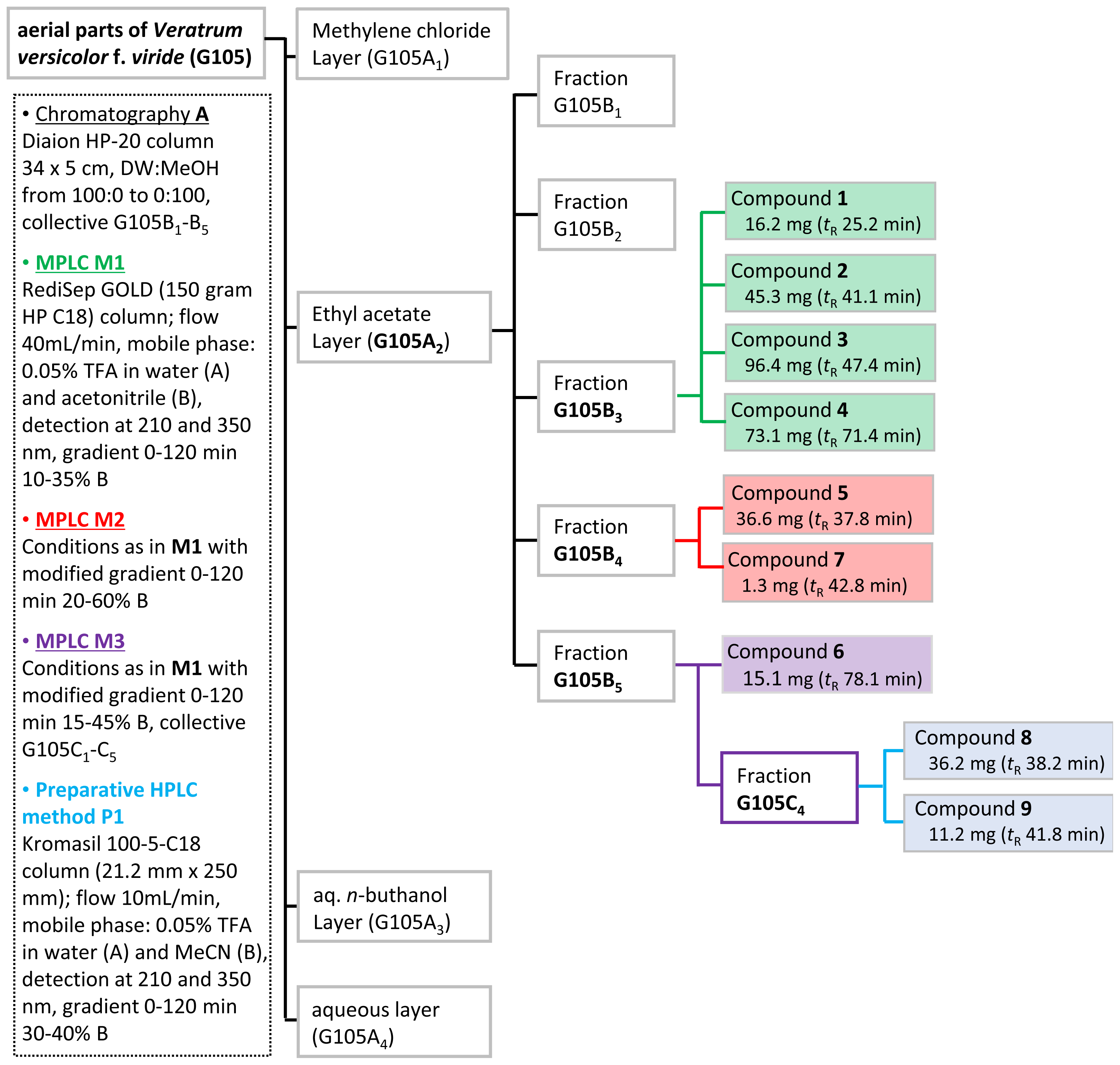

Supplement: Figure S1 — The isolation scheme of compounds 1–9. [file tjc-47-06-1346s1.tif]

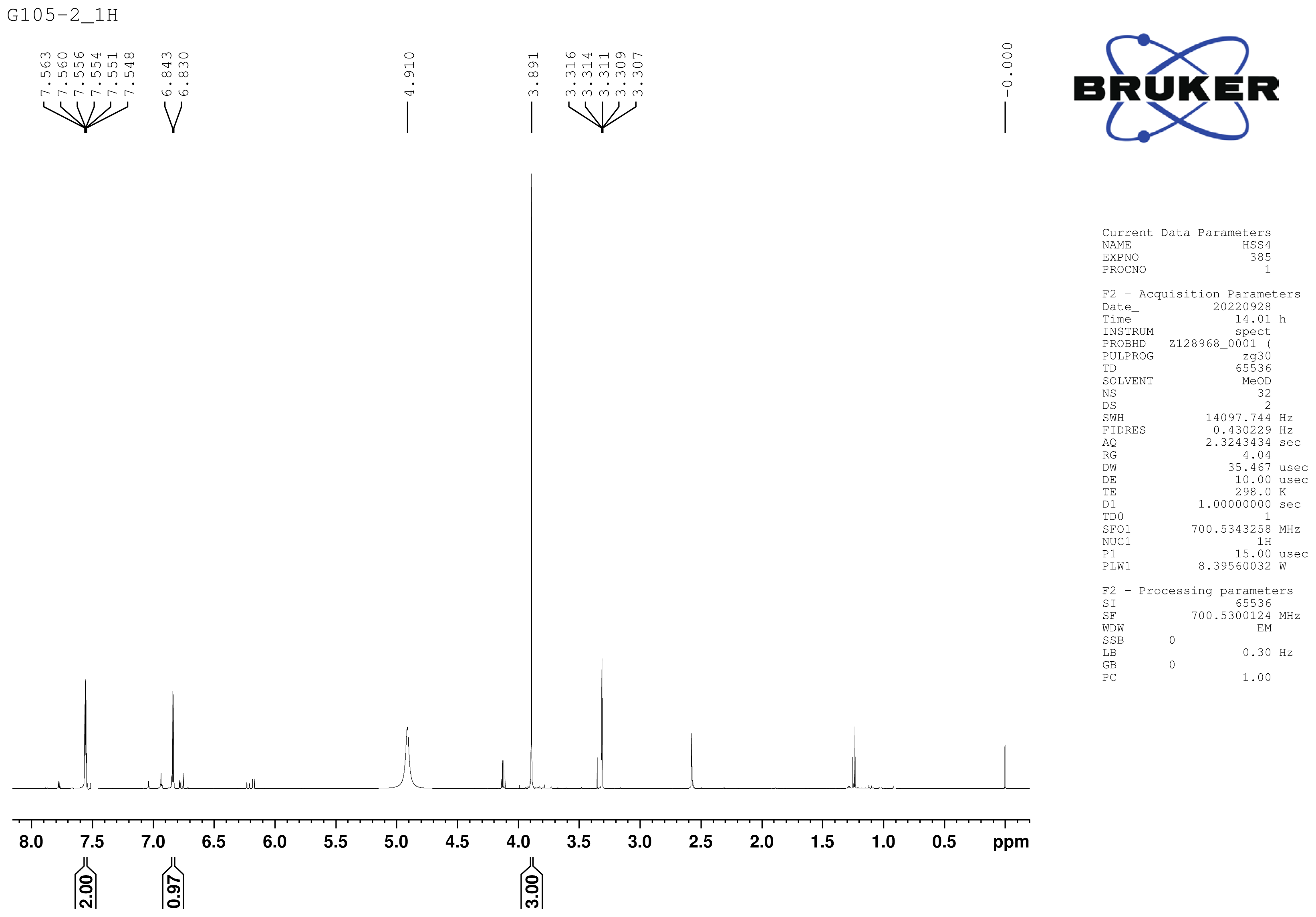

Supplement: Figure S2 — 1H NMR spectrum (CD3OD, 700 MHz) of compound 1. [file tjc-47-06-1346s2.tif]

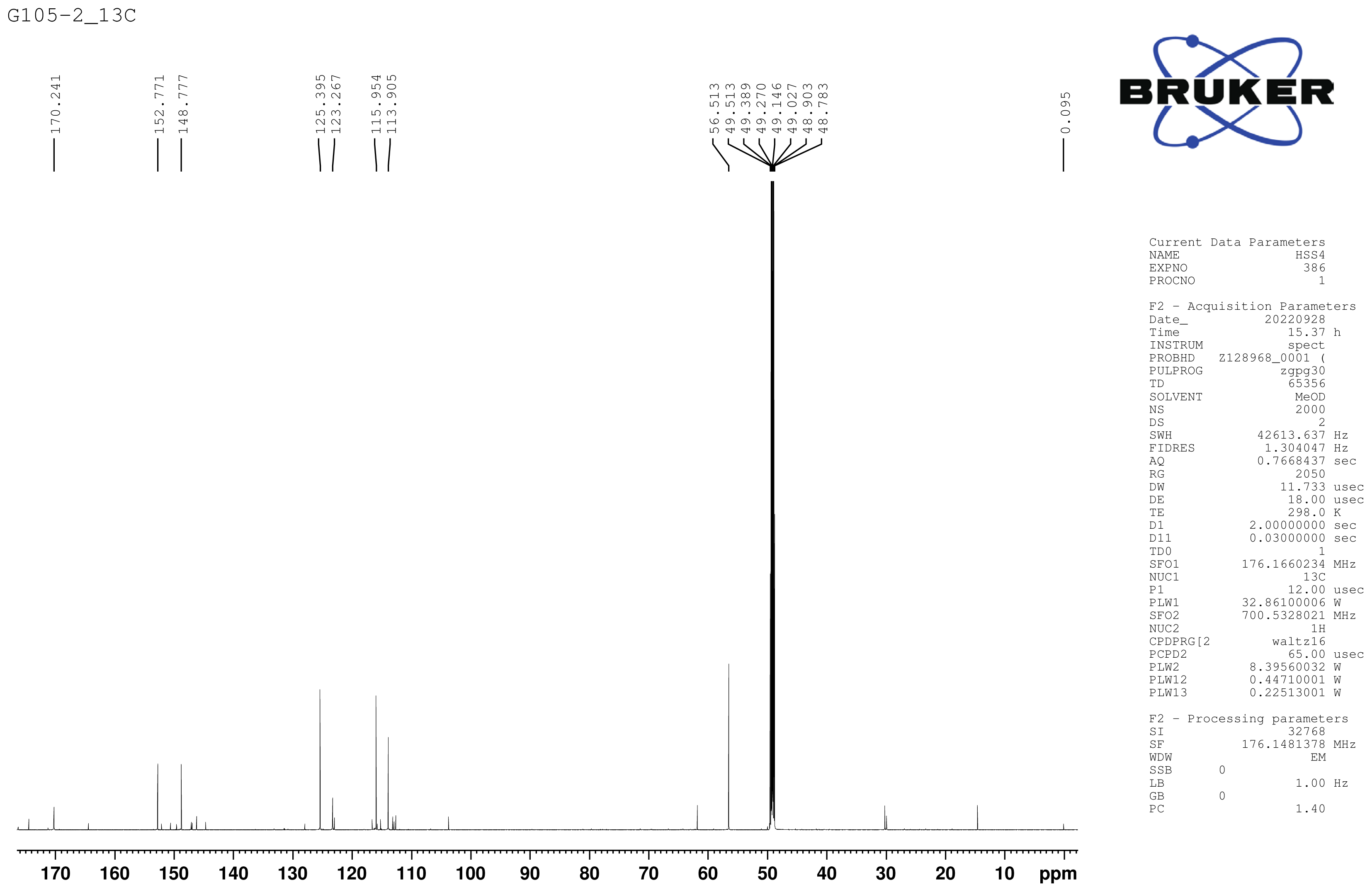

Supplement: Figure S3 — 13C NMR spectrum (CD3OD, 175 MHz) of compound 1. [file tjc-47-06-1346s3.tif]

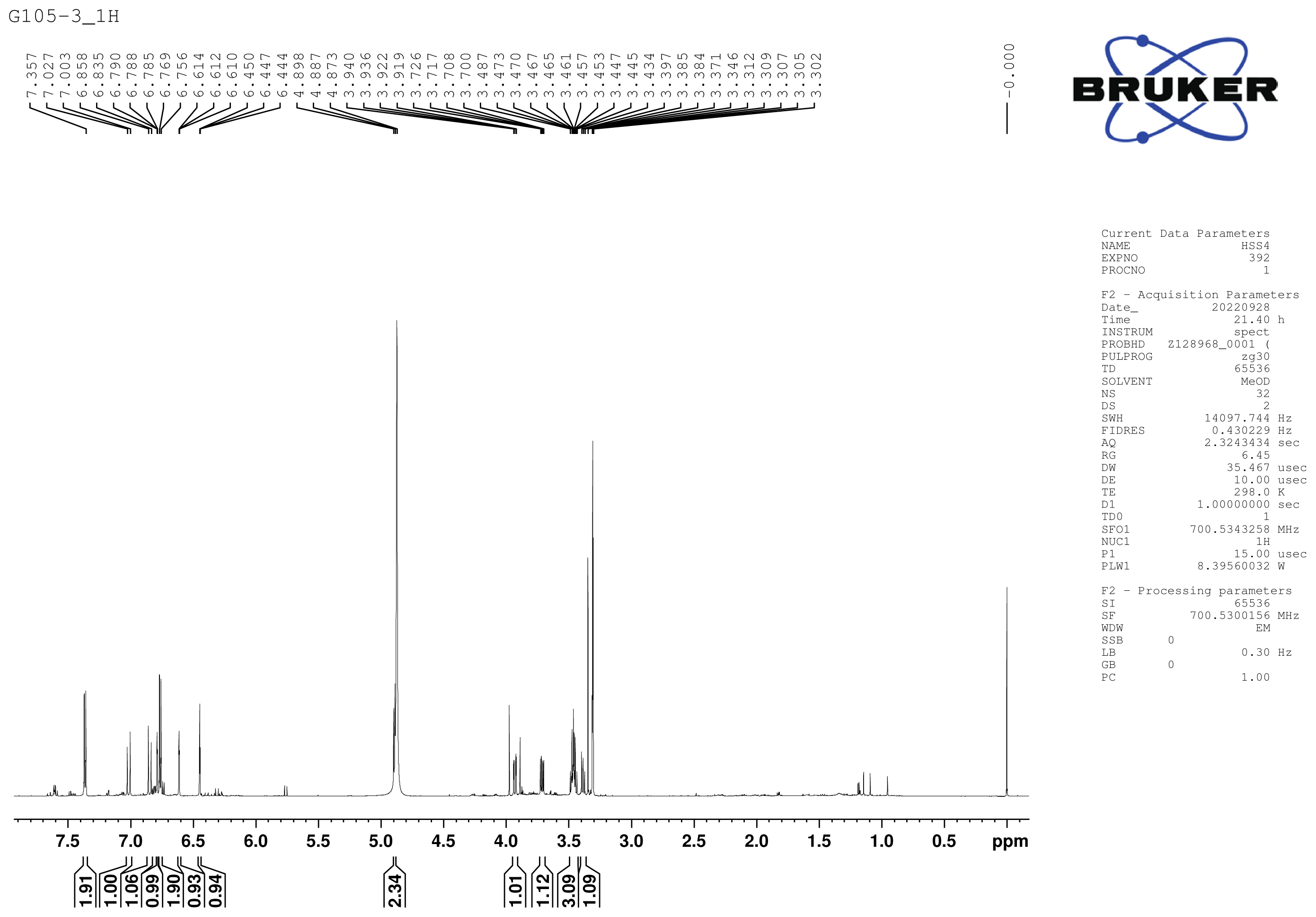

Supplement: Figure S4 — 1H NMR spectrum (CD3OD, 700 MHz) of compound 2. [file tjc-47-06-1346s4.tif]

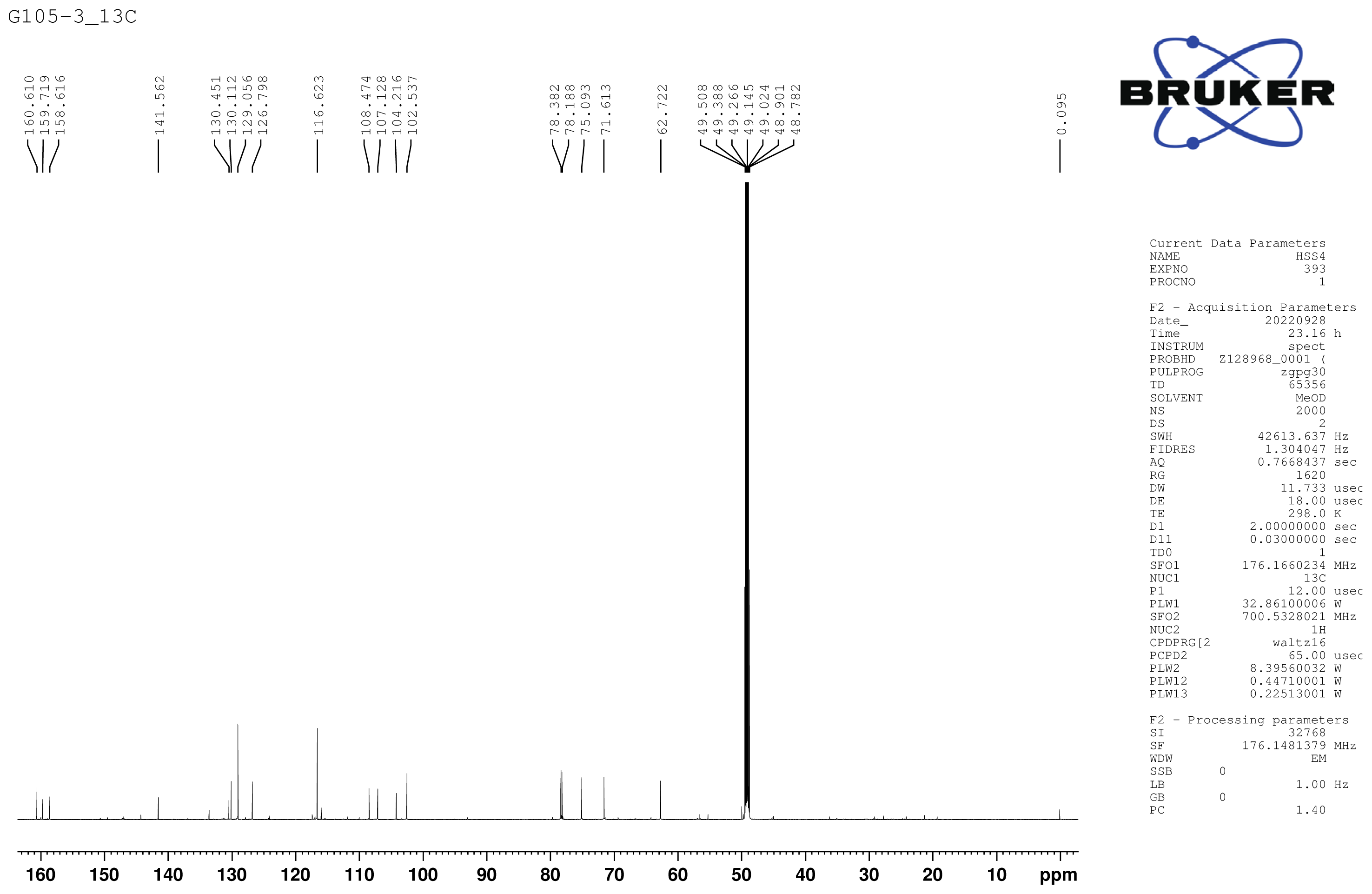

Supplement: Figure S5 — 13C NMR spectrum (CD3OD, 175 MHz) of compound 2. [file tjc-47-06-1346s5.tif]

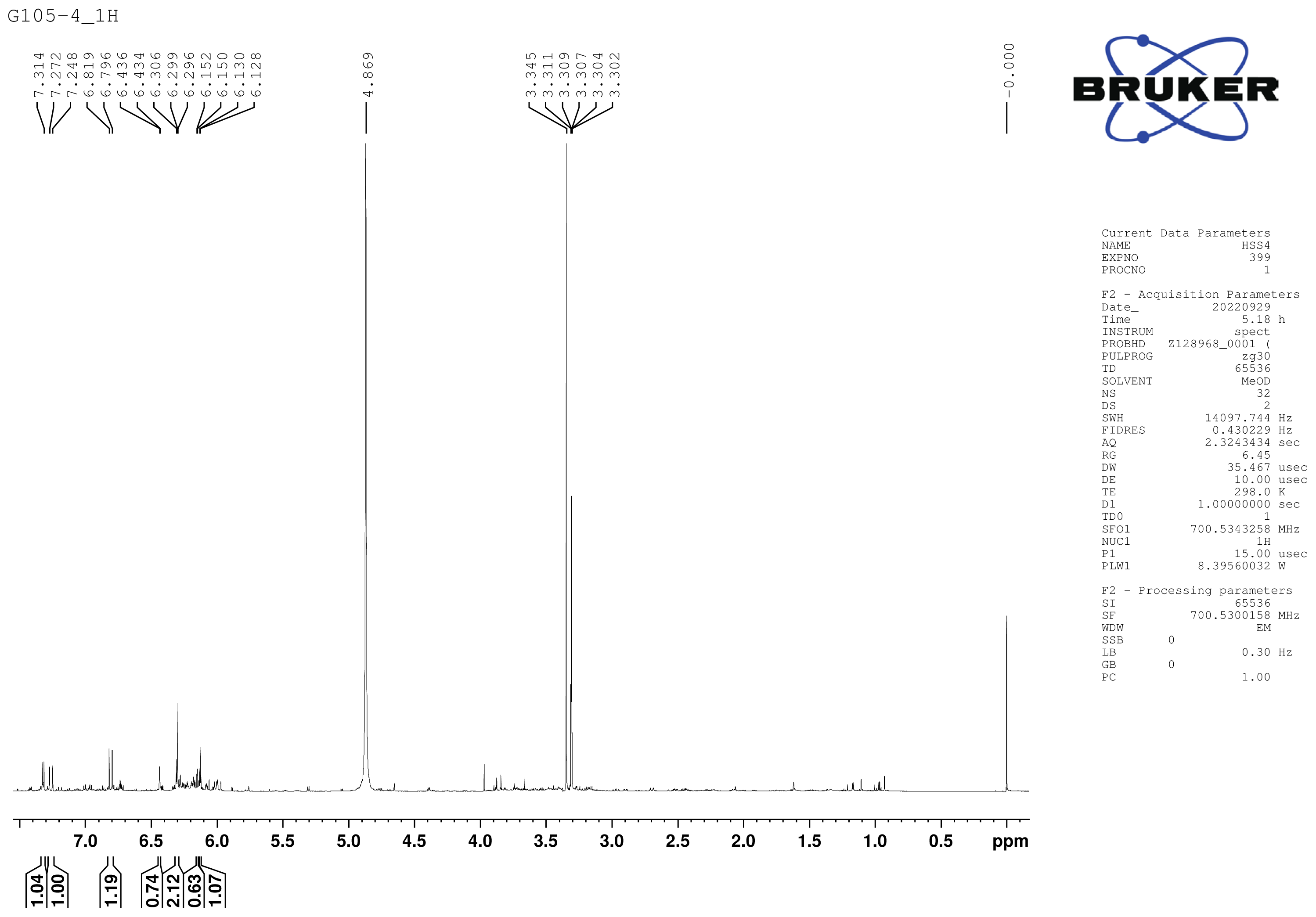

Supplement: Figure S6 — 1H NMR spectrum (CD3OD, 700 MHz) of compound 3. [file tjc-47-06-1346s6.tif]

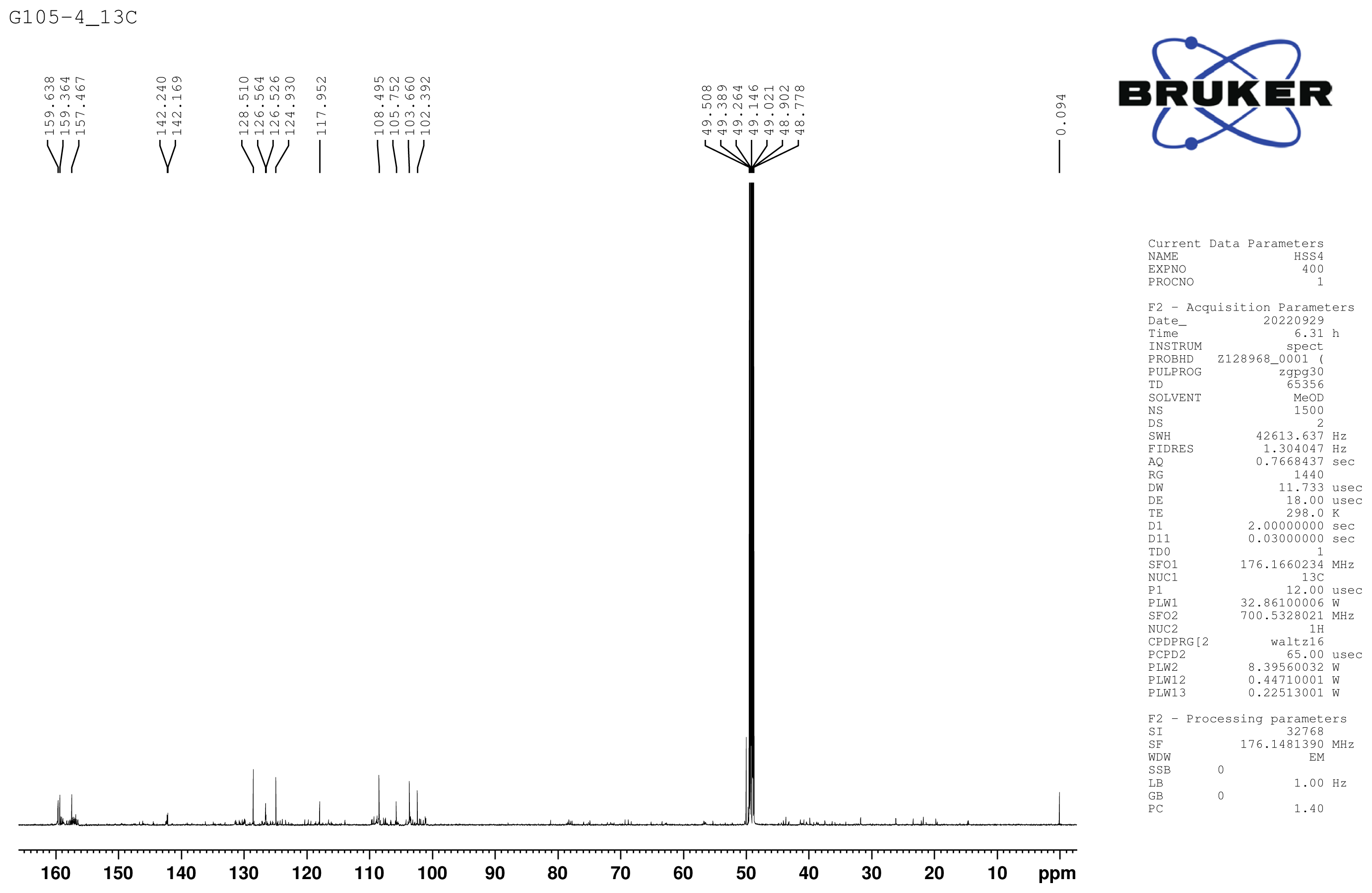

Supplement: Figure S7 — 13C NMR spectrum (CD3OD, 175 MHz) of compound 3. [file tjc-47-06-1346s7.tif]

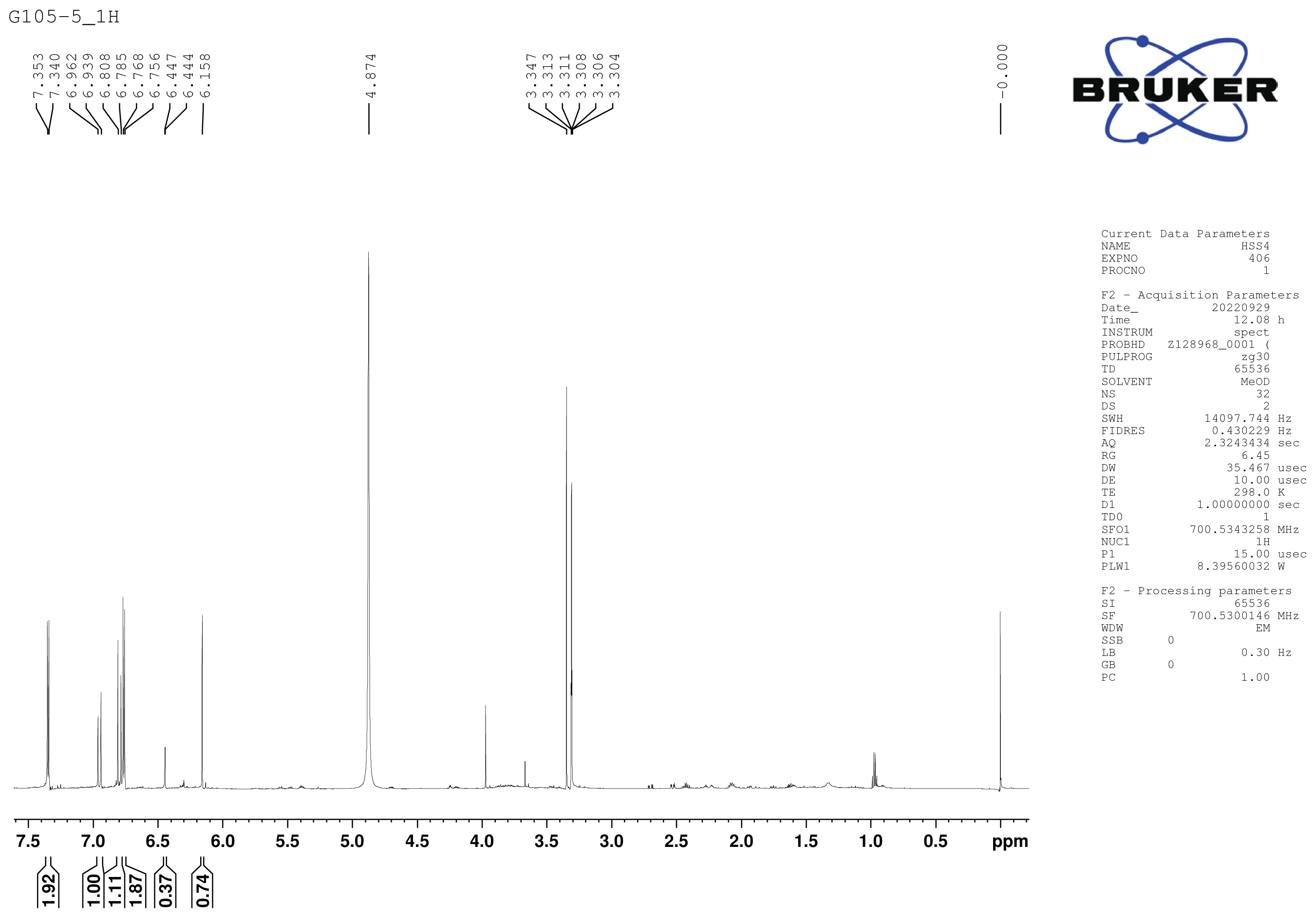

Supplement: Figure S8 — 1H NMR spectrum (CD3OD, 700 MHz) of compound 4. [file tjc-47-06-1346s8.tif]

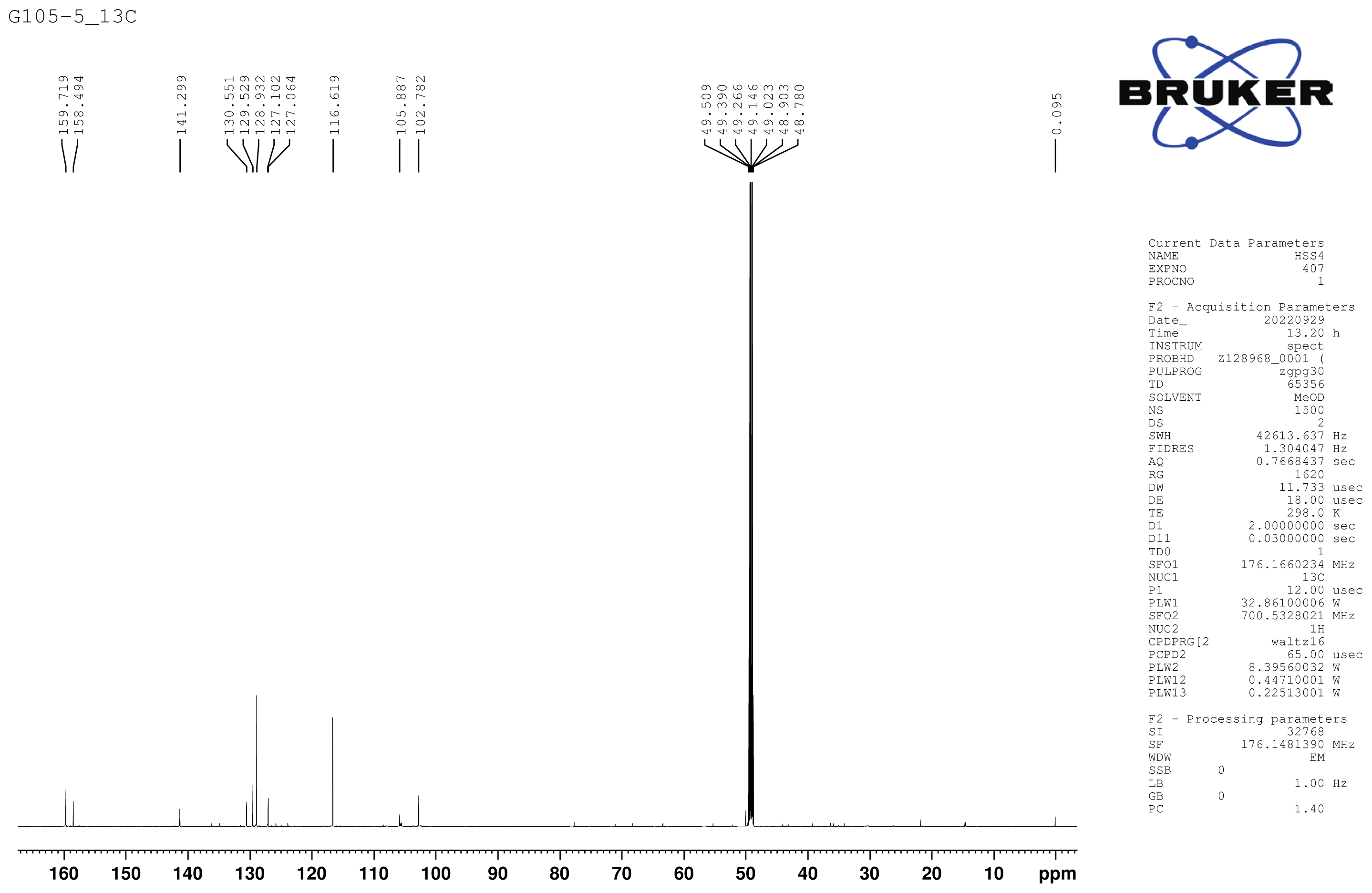

Supplement: Figure S9 — 13C NMR spectrum (CD3OD, 175 MHz) of compound 4. [file tjc-47-06-1346s9.tif]

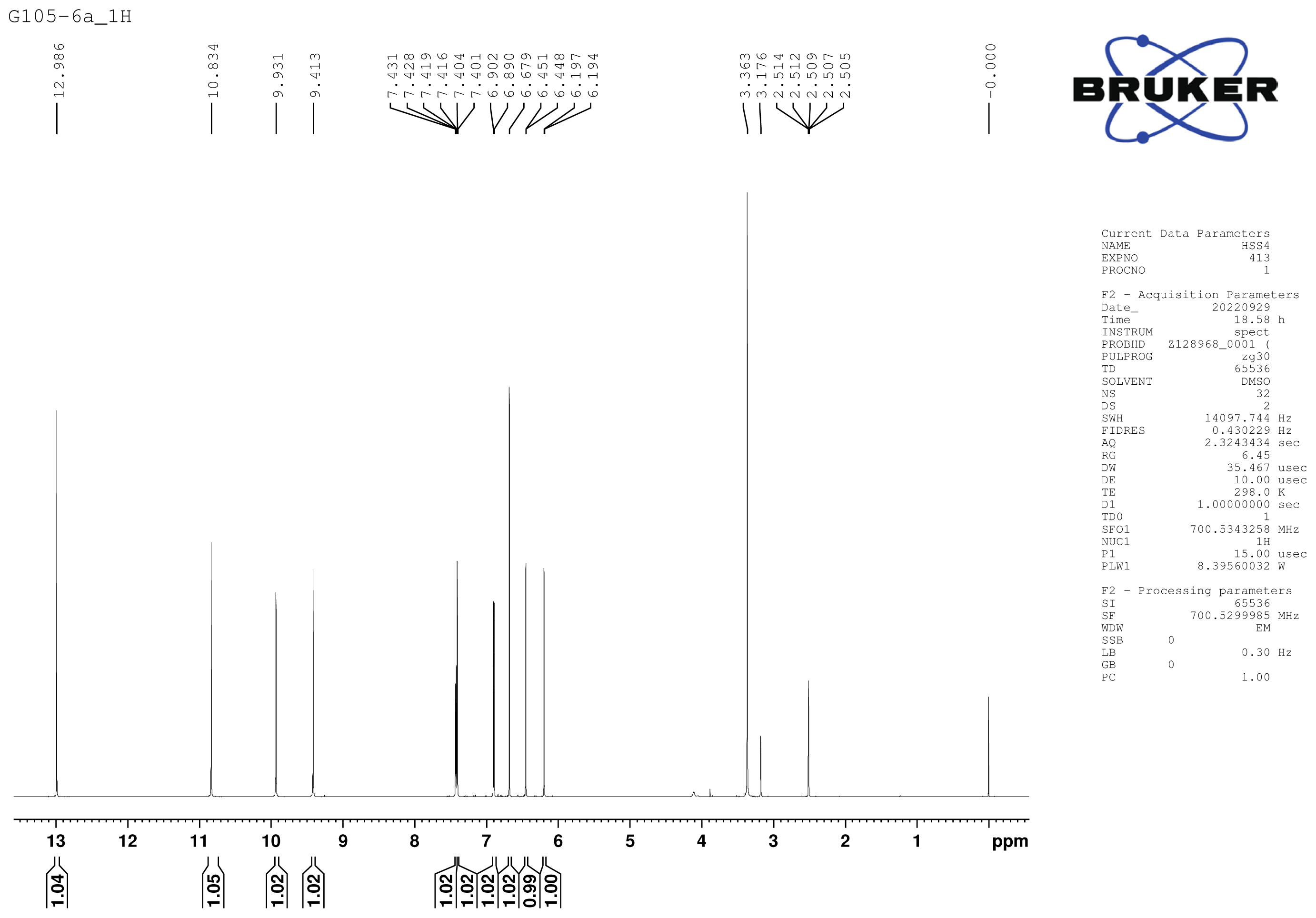

Supplement: Figure S10 — 1H NMR spectrum (DMSO-d6, 700 MHz) of compound 5. [file tjc-47-06-1346s10.tif]

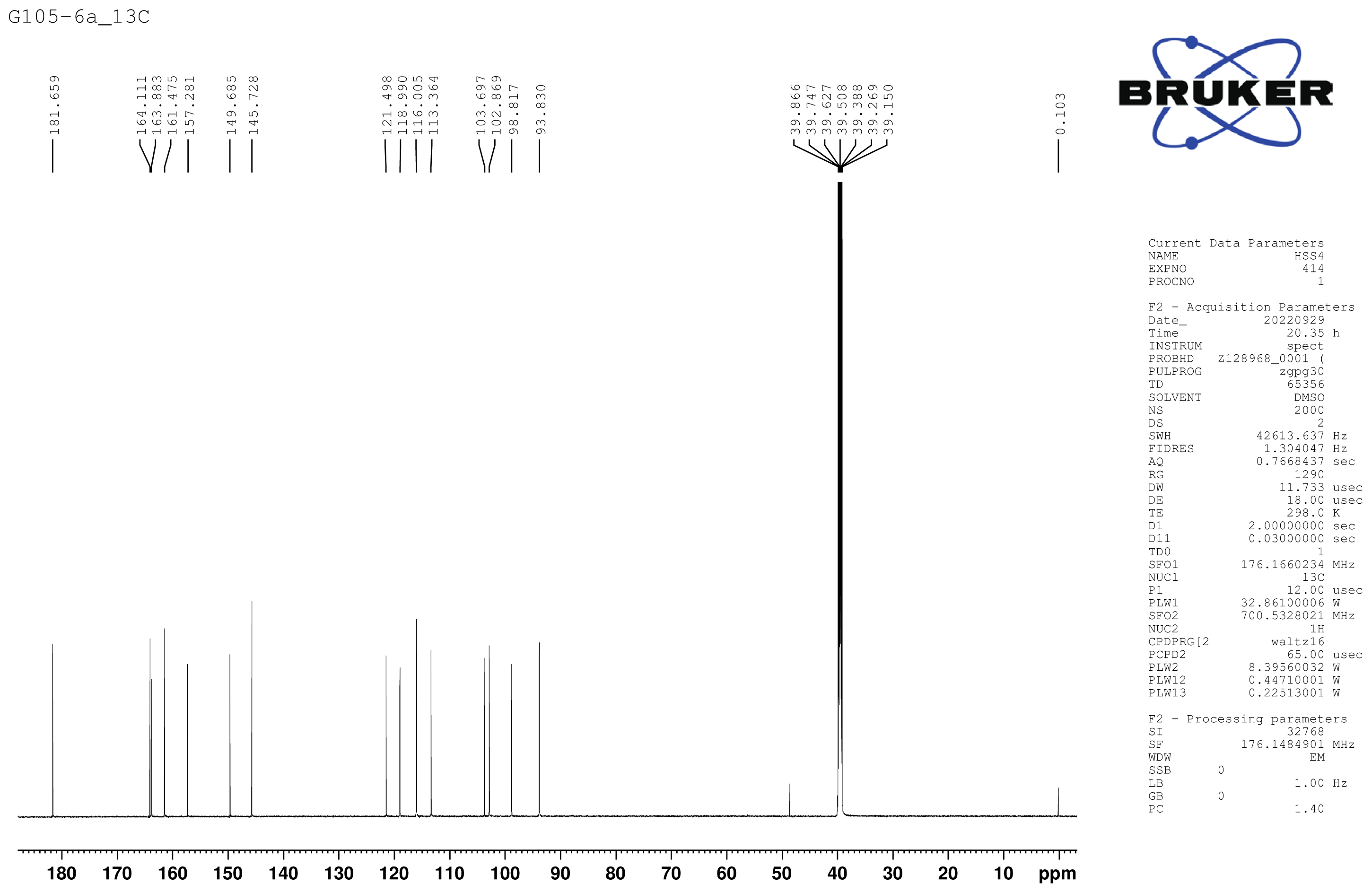

Supplement: Figure S11 — 13C NMR spectrum (DMSO-d6, 175 MHz) of compound 5. [file tjc-47-06-1346s11.tif]

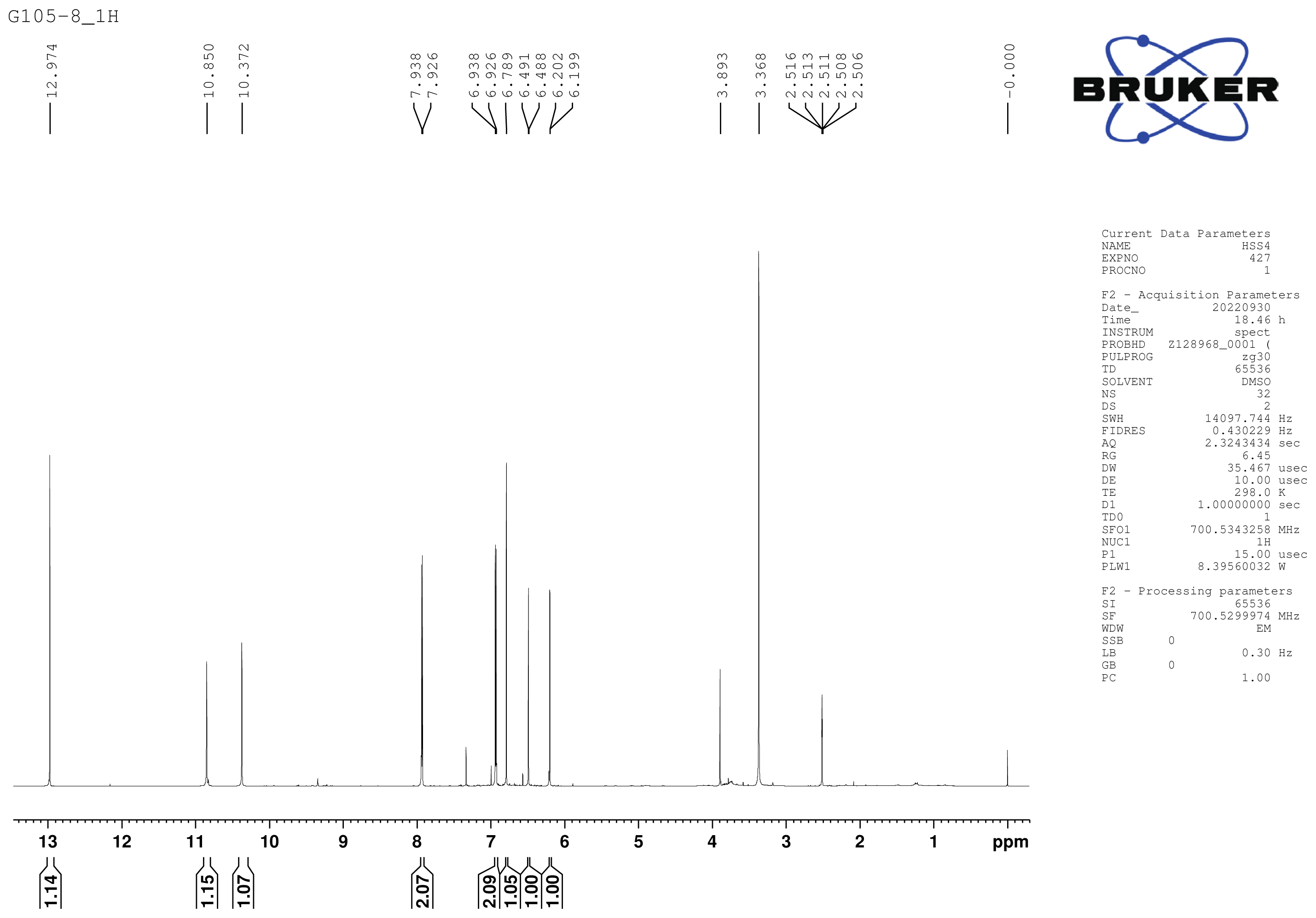

Supplement: Figure S12 — 1H NMR spectrum (DMSO-d6, 700 MHz) of compound 6. [file tjc-47-06-1346s12.tif]

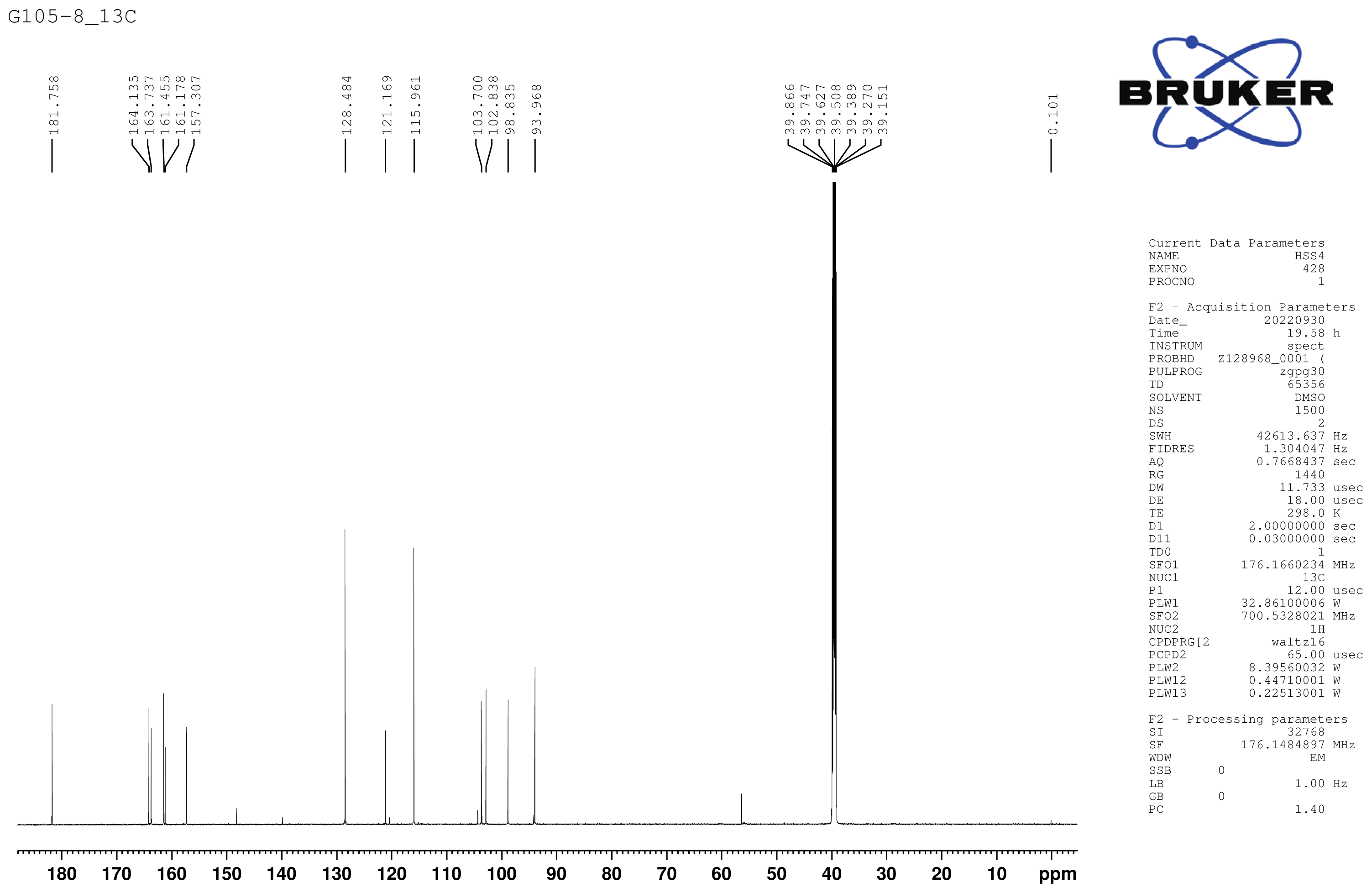

Supplement: Figure S13 — 13C NMR spectrum (DMSO-d6, 175 MHz) of compound 6. [file tjc-47-06-1346s13.tif]

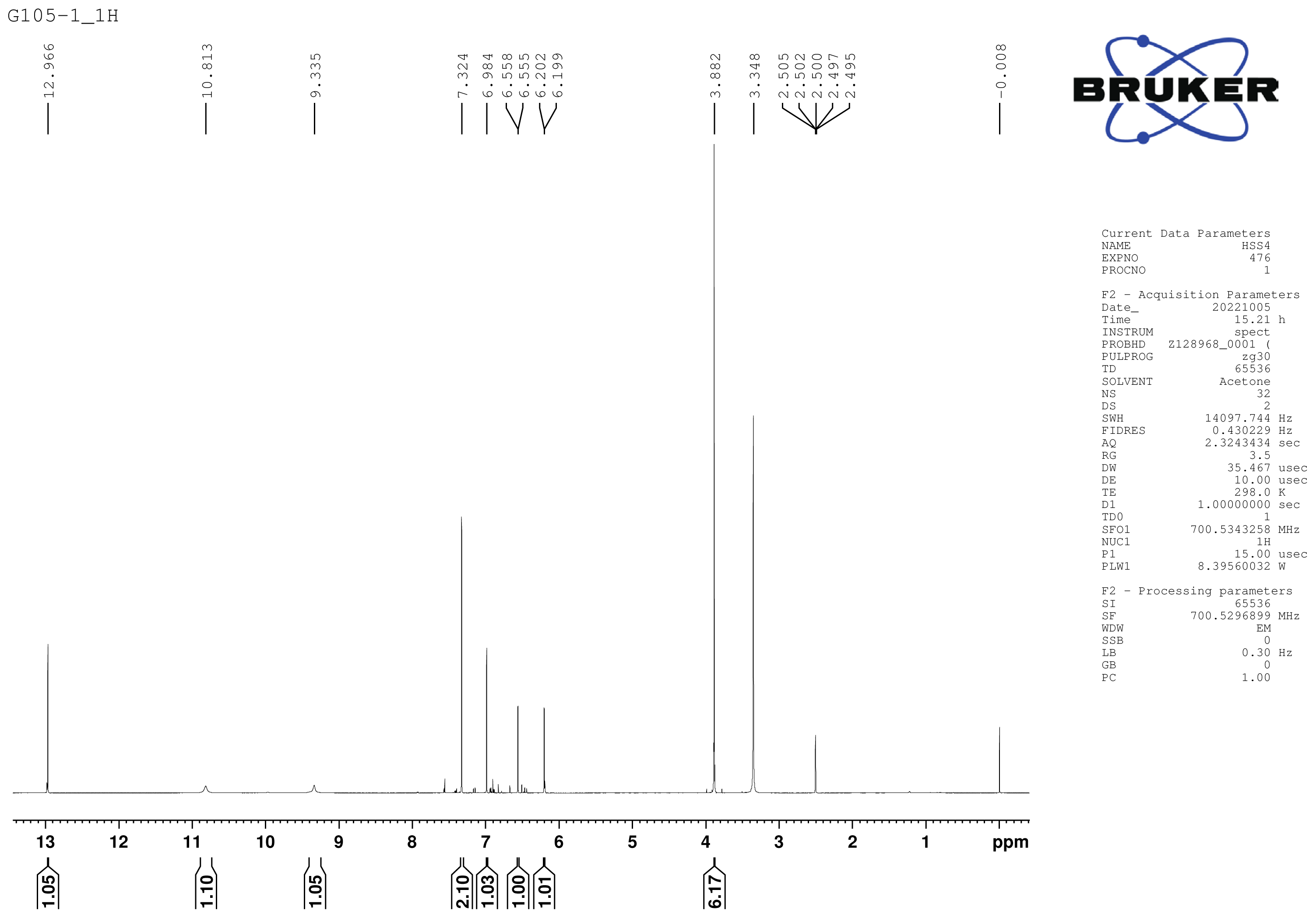

Supplement: Figure S14 — 1H NMR spectrum (DMSO-d6, 700 MHz) of compound 7. [file tjc-47-06-1346s14.tif]

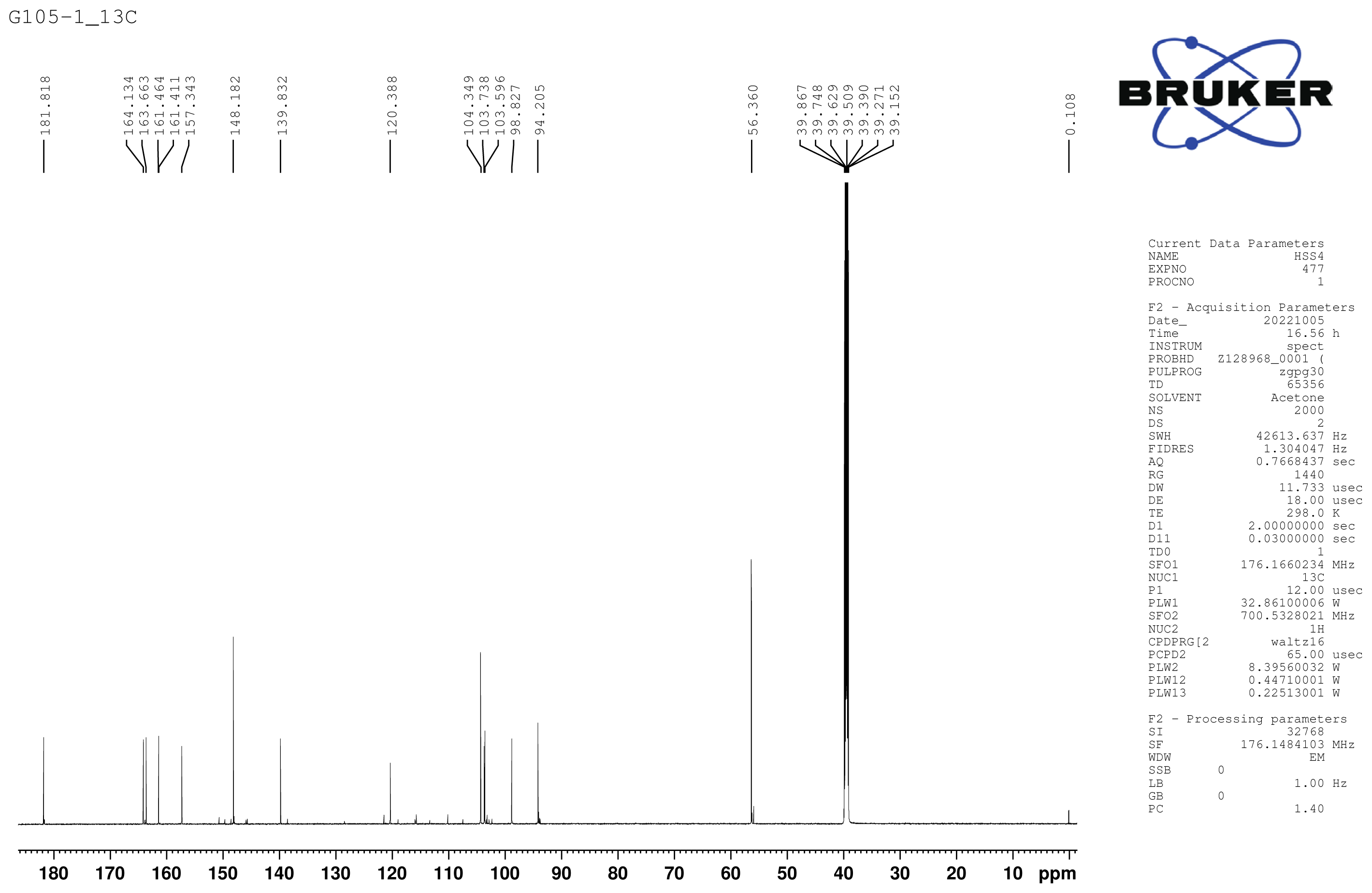

Supplement: Figure S15 — 13C NMR spectrum (DMSO-d6, 175 MHz) of compound 7. [file tjc-47-06-1346s15.tif]

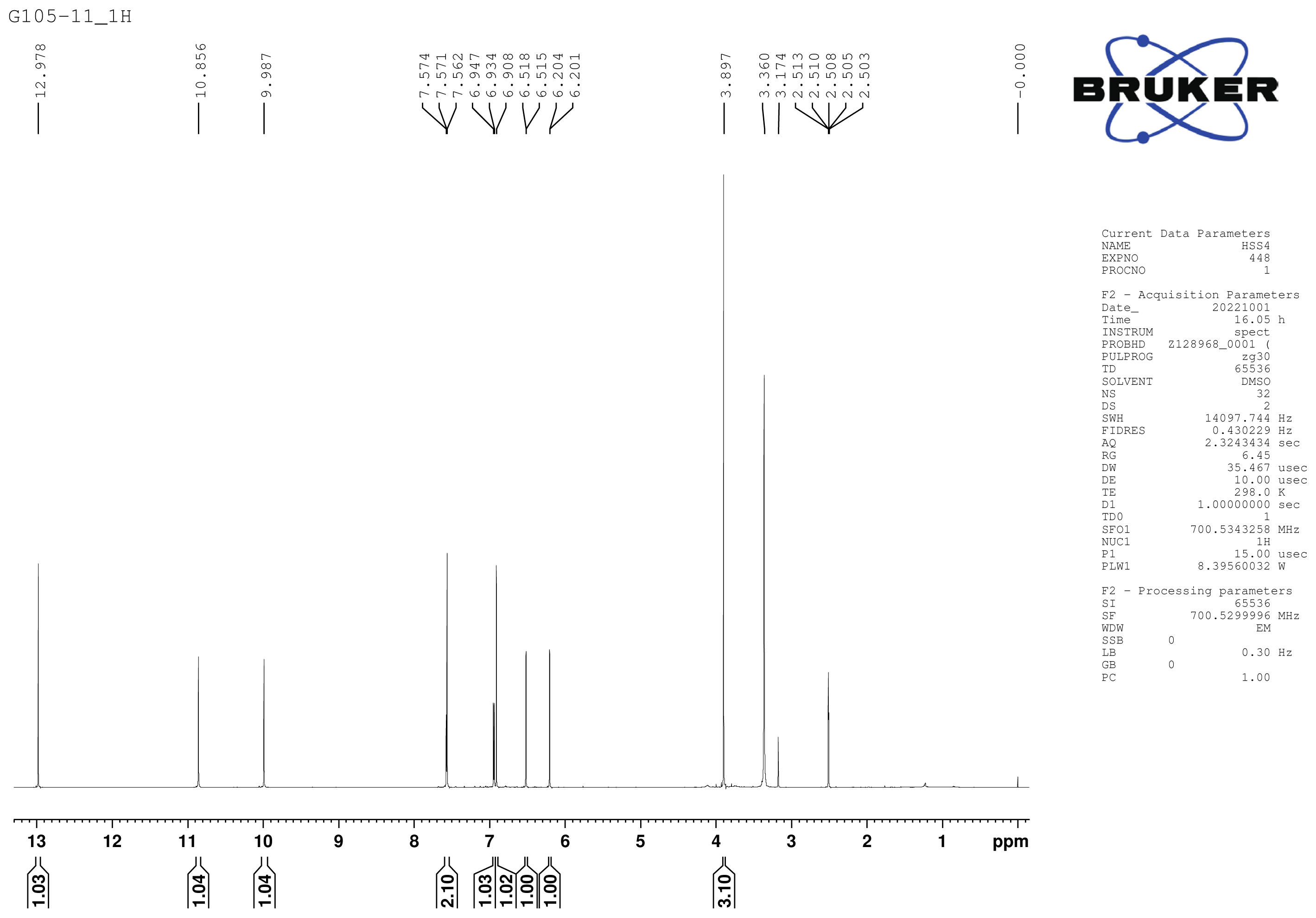

Supplement: Figure S16 — 1H NMR spectrum (DMSO-d6, 700 MHz) of compound 8. [file tjc-47-06-1346s16.tif]

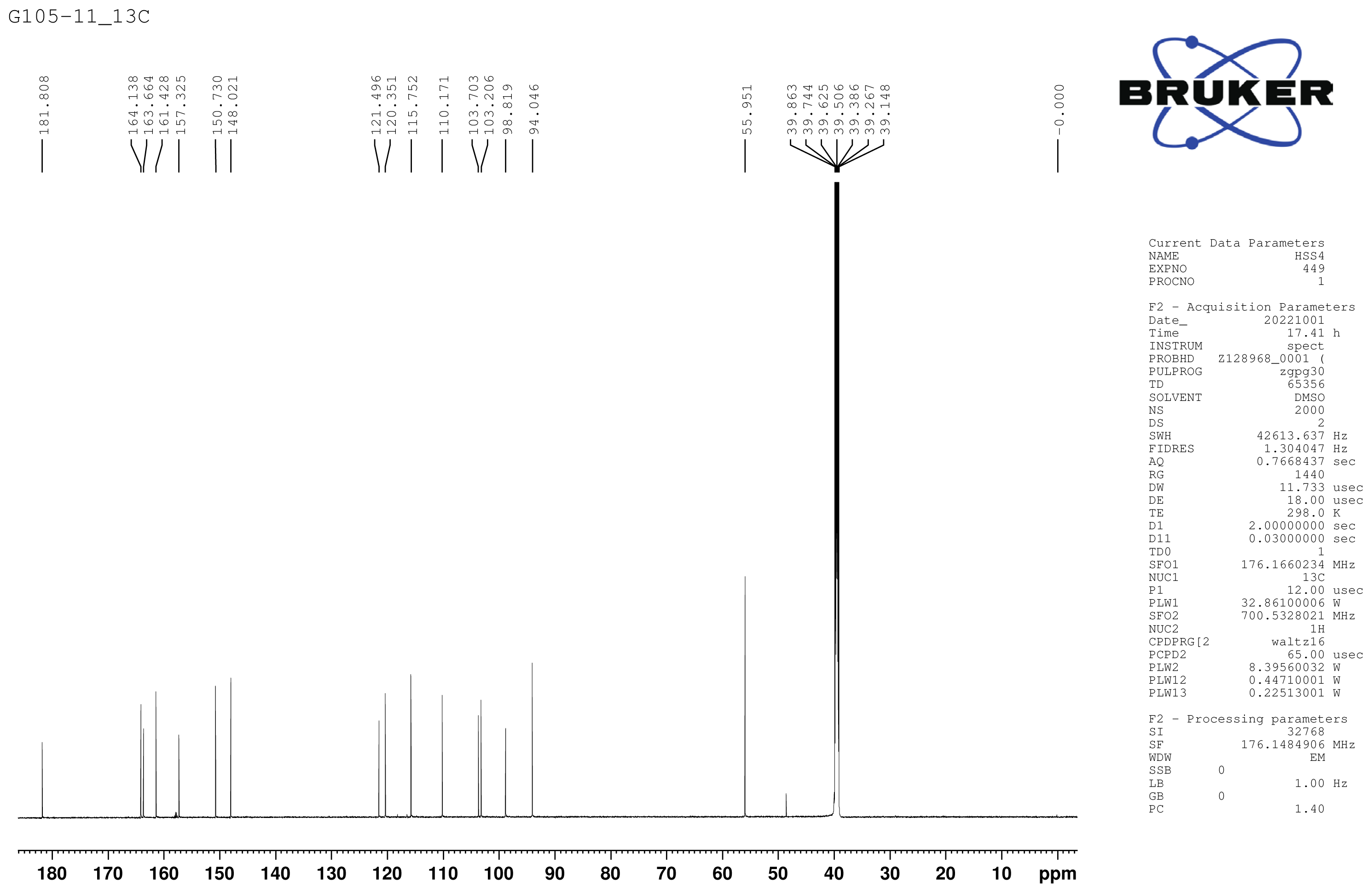

Supplement: Figure S17 — 13C NMR spectrum (DMSO-d6, 175 MHz) of compound 8. [file tjc-47-06-1346s17.tif]

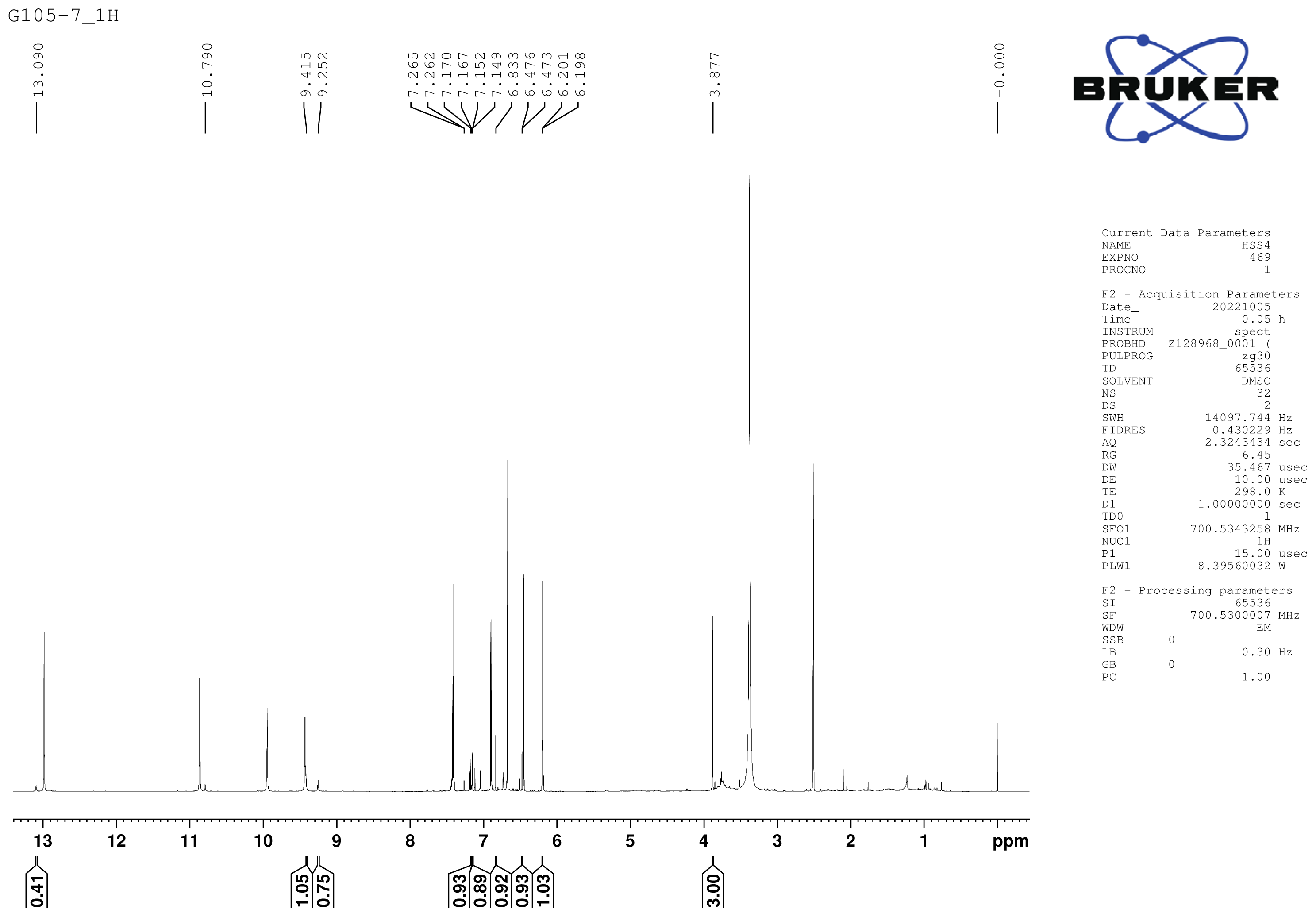

Supplement: Figure S18 — 1H NMR spectrum (DMSO-d6, 700 MHz) of compound 9. [file tjc-47-06-1346s18.tif]

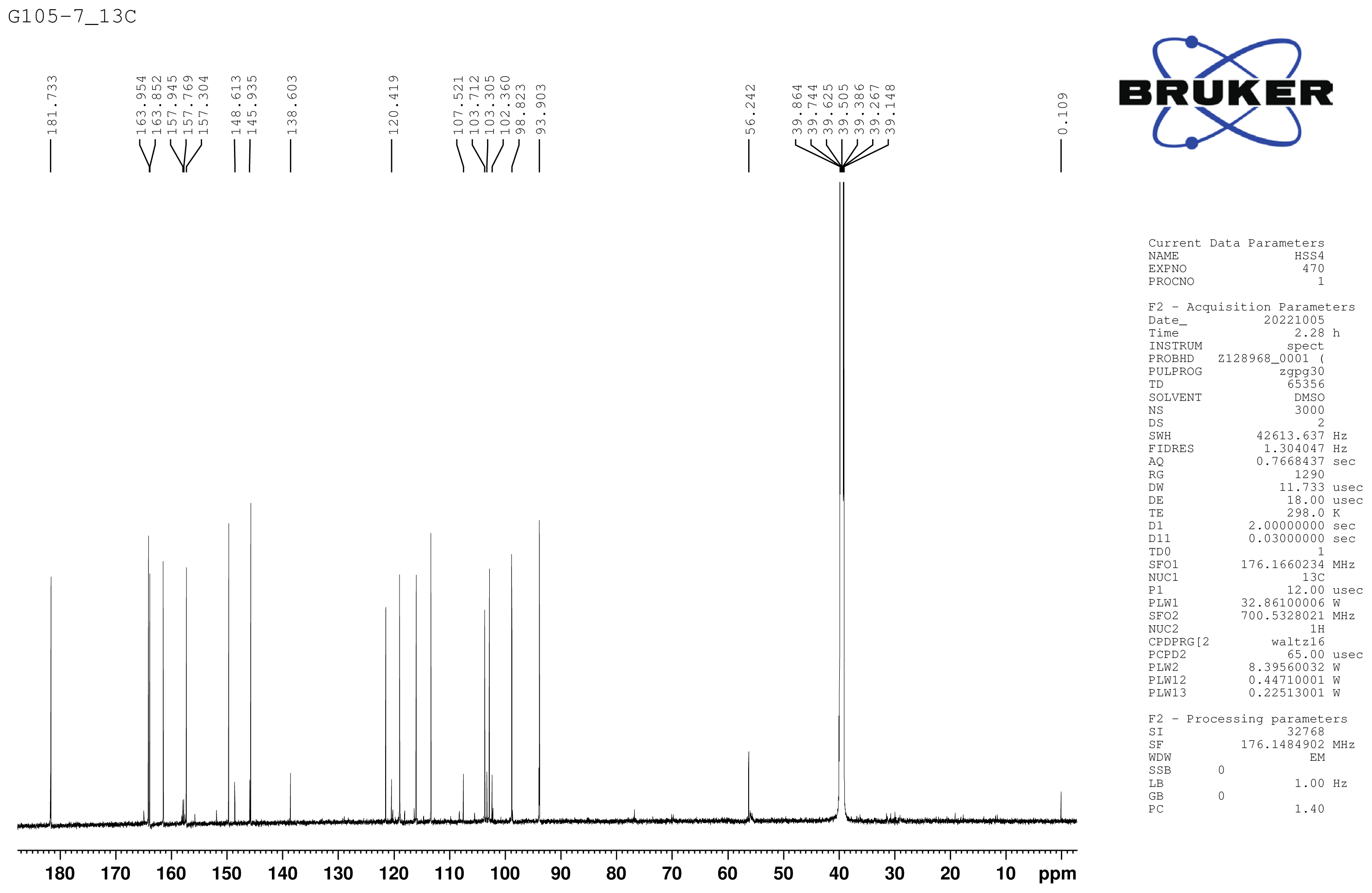

Supplement: Figure S19 — 13C NMR spectrum (DMSO-d6, 175 MHz) of compound 9. [file tjc-47-06-1346s19.tif]
